# Ameliorative Effect of *Ocimum forskolei* Benth on Diabetic, Apoptotic, and Adipogenic Biomarkers of Diabetic Rats and 3T3-L1 Fibroblasts Assisted by In Silico Approach

**DOI:** 10.3390/molecules27092800

**Published:** 2022-04-28

**Authors:** Hany Ezzat Khalil, Miada F. Abdelwahab, Promise Madu Emeka, Lorina I. Badger-Emeka, Krishnaraj Thirugnanasambantham, Hairul-Islam Mohamed Ibrahim, Sara Mohamed Naguib, Katsuyoshi Matsunami, Nada M. Abdel-Wahab

**Affiliations:** 1 Department of Pharmaceutical Sciences, College of Clinical Pharmacy, King Faisal University, Al-Ahsa 31982, Saudi Arabia; pemeka@kfu.edu.sa; 2 Department of Pharmacognosy, Faculty of Pharmacy, Minia University, Minia 61519, Egypt; mayada.mohamed2@mu.edu.eg (M.F.A.); nada.abdelwahab@mu.edu.eg (N.M.A.-W.); 3 Department of Biomedical Sciences, College of Medicine, King Faisal University, Al-Ahsa 31982, Saudi Arabia; lbadgeremeka@kfu.edu.sa; 4 Pondicherry Centre for Biological Science and Educational Trust, Kottakuppam 605104, India; researchdirector@pcbsindia.com (K.T.); himohamed@kfu.edu.sa (H.-I.M.I.); 5 Department of Biological Sciences, College of Science, King Faisal University, Al-Ahsa 31982, Saudi Arabia; 6 Department of Histology and Cell Biology, Faculty of Medicine, Minia University, Minia 61111, Egypt; sara.abdelhafez@mu.edu.eg; 7 Department of Pharmacognosy, Graduate School of Biomedical & Health Sciences, Hiroshima University, 1-2-3 Kasumi, Minami-ku, Hiroshima 734-8553, Japan; matunami@hiroshima-u.ac.jp

**Keywords:** *Ocimum forskolei*, hesperidin, streptozotocin, diabetes, 3T3-L1, NF-κB, PPARγ

## Abstract

Diabetes mellitus (DM) is a complicated condition that is accompanied by a plethora of metabolic symptoms, including disturbed serum glucose and lipid profiles. Several herbs are reputed as traditional medicine to improve DM. The current study was designed to explore the chemical composition and possible ameliorative effects of *Ocimum forskolei* on blood glucose and lipid profile in high-fat diet/streptozotocin-induced diabetic rats and in 3T3-L1 cell lines as a first report of its bioactivity. Histopathological study of pancreatic and adipose tissues was performed in control and treatment groups, along with quantification of glucose and lipid profiles and the assessment of NF-κB, cleaved caspase-3, BAX, and BCL2 markers in rat pancreatic tissue. Glucose uptake, adipogenic markers, DGAT1, CEBP/α, and PPARγ levels were evaluated in the 3T3-L1 cell line. Hesperidin was isolated from total methanol extract (TME). TME and hesperidin significantly controlled the glucose and lipid profile in DM rats. Glibenclamide was used as a positive control. Histopathological assessment showed that TME and hesperidin averted necrosis and infiltration in pancreatic tissues, and led to a substantial improvement in the cellular structure of adipose tissue. TME and hesperidin distinctly diminished the mRNA and protein expression of NF-κB, cleaved caspase-3, and BAX, and increased BCL2 expression (reflecting its protective and antiapoptotic actions). Interestingly, TME and hesperidin reduced glucose uptake and oxidative lipid accumulation in the 3T3-L1 cell line. TME and hesperidin reduced DGAT1, CEBP/α, and PPARγ mRNA and protein expression in 3T3-L1 cells. Moreover, docking studies supported the results via deep interaction of hesperidin with the tested biomarkers. Taken together, the current study demonstrates *Ocimum forskolei* and hesperidin as possible candidates for treating diabetes mellitus.

## 1. Introduction

Diabetes mellitus (DM) is a complex, chronic illness that is characterized by metabolic disorders such as high blood glucose levels. Three major types of diabetes have been identified: type 1, related to insulin deficiency due to pancreatic β cell destruction; type 2 (90% of patients), associated with insulin resistance and insulin secretion deficiency; and gestational diabetes, developed during pregnancy, leading to complications and increasing the risk of type 2 diabetes in the mother [1,2]. The risk factors for type 1 diabetes are not known; however, a complicated interplay between genetic and environmental factors is involved in its etiology. For type 2 diabetes, obesity represents one of the most potent risk factors, followed by age, low physical activity, family history, ethnicity, and smoking. The risk factors for developing gestational diabetes are similar to those for type 2 diabetes, with the additional risk of excessive weight gain during pregnancy [1,2,3]. Unmonitored diabetes leads to serious complications, typically described as microvascular (nephropathy, neuropathy, and retinopathy) and macrovascular (cardiovascular), in addition to other attributable complications, including infections, cancer, depressive disorders, dementia, disability, and death [4].

Despite the fact that synthetic drugs such as sulfonylureas, dipeptidyl peptidase-4 inhibitors, thiazolidinedione, biguanides, and α-glucosidase inhibitors demonstrate therapeutic benefits and effectiveness in the treatment of diabetes, they can produce many undesirable side effects in the long term [5]. Studies investigating the effect of natural products play a pivotal role in the discovery of new phytoactive compounds that are effective against several diseases. The huge demand for natural antidiabetic products and herbs is attributed to their multiple constituents, multiple targets, and lower toxicity, which may overcome the disadvantages of presently used therapies [5,6]. Due to these advantages, recent studies have shown that more natural products are currently being explored for better management of diabetes, especially type 2 [7,8]. Numerous common herbs used in traditional medicine are reported to lower blood glucose level via different mechanisms. Such herbs include *Cinnamomum zeylanicum* bark, *Ficus racemosa* bark, *Nigella sativa* seeds, *Ocimum basilicum* leaves, *Aloe barbadensis* leaves, *Trigonella foenum-graecum* seeds, *Cuminum cyminum* fruits, *Panax ginseng*, and *Allium sativum* [9]. In addition, polyherbal formulations are available on the market as favorable adjuvant and alternative therapies for diabetes mellitus to take advantage of synergistic or additive effects [8,9,10].

Evaluation of herbal antidiabetic medicines has indicated that the metabolites responsible for their activity are predominantly polysaccharides, flavonoids, polyphenols, terpenoids, alkaloids, saponins, and quinones [5,11]. Lamiaceae is a flowering plant family that encompasses 236 genera with approximately 7136 species. A wide variety of these herbaceous plants exhibit important economic, biologic, and medicinal applications [12,13]. Lamiaceae species possess a wide range of bioactivity, namely, antimicrobial, antiseptic, antispasmodic, carminative, analgesic, and antidiabetic [13,14]. *Ocimum*, in the subfamily Nepetoideae and incorporating about 160 species, is one of the most important genera in this family [15]. Studies investigating the biological activity of various *Ocimum* species revealed antidiabetic activity in *Ocimum tenuiflorum* [16,17], *Ocimum gratissimum* [18], and *Ocimum basilicum* [19].

*Ocimum forskolei* Benth (*O. forskolei*) is an aromatic herb traditionally used as a flavoring agent in Saudi Arabia, insect repellant in Eretria, antipyretic in Yemen, and for treating eye infection in Rwanda [20,21]. Previous studies on this plant have demonstrated that it has several biological activities, such as local anesthetic [22], antiepileptic [20], antiulcer [23,24], and anti-inflammatory effects [25]. In addition, it possesses antimicrobial, antioxidant, and cytotoxic activities [21]. Interestingly, a recent in vitro study on *O. forskolei*, conducted with methanol extract of its leaves and stems, showed considerable antidiabetic results through inhibition of α-amylase and glycosylation of hemoglobin [26].

Considering this background, the present work was conducted to evaluate, for the first time, the potential hypoglycemic and hypolipidemic activities of *O. forskolei*, as well as the isolated flavonoid hesperidin. The study was supported by histopathological investigation. The results were confirmed using the 3T3-L1 cell line in vitro, as well as in silico studies. The current study is an attempt to explore a bioactive natural candidate to treat diabetes with fewer adverse effects, which may prove favorable to synthetic drugs.

## 2. Results

### 2.1. Isolation and Identification of Hesperidin

**Figure 1:**
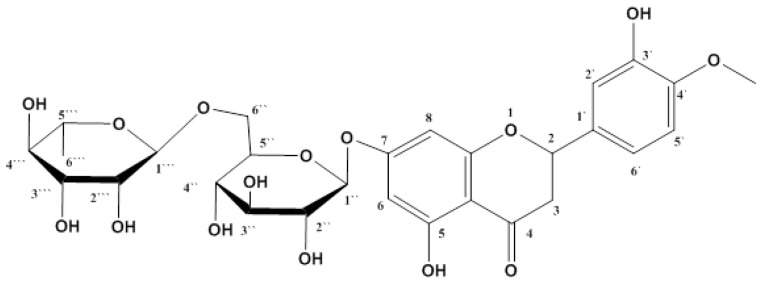
Structure of isolated pure hesperidin from TME of *O. forskolei*.

The total methanol extract (TME) of *O. forskolei* (500 g) was subjected to several repeated chromatographic techniques to yield the pure flavonoid compound hesperidin (HSP) (1.3 g). The structure was elucidated by 1D- and 2D-NMR spectroscopy and compared with the literature values and authentic HSP (Supplementary Materials, Supplementary S1) [27]. This study represents the first report on the isolation of HSP (Figure 1) from *O. forskolei*. Hesperidin: ^1^H-NMR (DMSO-*d*6, 400 MHz) δ: 12.04 (1H, br s, 5-OH), 6.95 (1H, m, H-2′), 6.94(1H, m, H-5′), 6.92 (1H, m, H-6′), 6.16 (1H, s, H-8), 6.14 (1H, s, H-6), 5.50 (1H, br d, J = 11.4 Hz, H-2), 4.97 (1H, d, J = 6.7 Hz, H-1″), 4.54 (1H, br s, H-1‴), 3.78 (3H, s, 4′-OCH_3_), 3.25–3.65 (6H, m, H-2″ to H-6″), 3.25–3.65 (3H, m, H-2‴ to H-6‴), 3.17 (1H, br s, H-3a), 2.78 (1H, br d, J = 17.1 Hz, H-3b), 2.51 (1H, br s, H-5‴), 1.09 (3H, d, J = 6.2 Hz, H-6‴); ^13^C -NMR (DMSO-*d*6, 100 MHz) δ: 197.0 (s, C-4), 165.1 (s, C-7), 163.0 (s, C-5), 162.4 (s, C-9), 147.9 (s, C-3′), 146.4 (s, C-4′), 130.8 (s, C-1′), 117.9 (s, C-6′), 114.1 (d, C-2′), 112.0 (d, C-5′), 103.3 (s, C-10), 100.5 (d, C-1‴), 99.4 (d, C-1″), 96.3 (d, C-6), 95.5 (d, C-8), 78.3 (d, C-2), 76.2 (d, C-3″), 75.5 (d, C-4″), 72.9 (d, C-2″), 72.0 (d, C-4‴), 70.7 (d, C-4″), 70.2 (d, C-3‴), 69.6 (d, C-2‴), 68.3 (d, C-5‴), 66.0 (t, C-6‴), 55.6 (q, 4-OCH_3_), 42.0 (t, C-3), 17.8 (q, C-6‴).

### 2.2. Effect of TME and HSP on Blood Glucose Level

**Figure 2:**
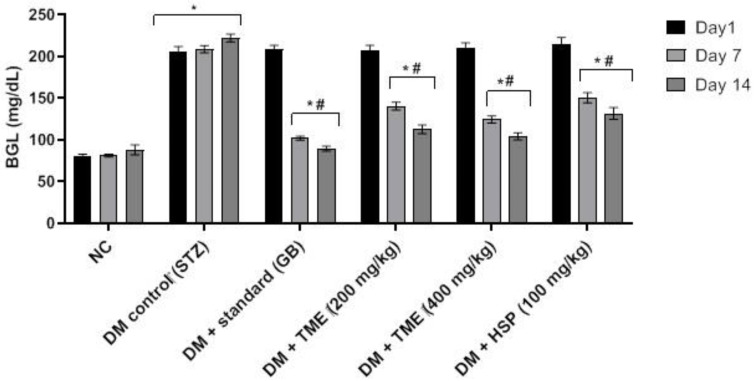
The effect of TME (200 and 400 mg/kg) and HSP (100 mg/kg) on STZ + HFD-induced diabetes. Results are presented as mean ± SD (*n* = 6); * significant difference between control group and all the other treatment groups; **#** significant difference between diabetic nontreated group and all treatment groups (TME and HSP). NC, normal control; TME, total methanol extract; HSP, hesperidin; STZ, streptozotocin; GB, glibenclamide; DM, diabetes mellitus; BGL, blood glucose level.

Injection of streptozotocin (STZ) at 35 mg/kg after feeding with a high-fat diet (HFD) showed a significant (*p* < 0.001) increase in blood glucose level (BGL) when compared with control rats (Figure 2). Day 1 before treatment revealed significantly (*p* < 0.05) increased blood glucose levels in all groups compared to the control group (NC). However, day 7 after treatment with glibenclamide (GB, standard drug), TME (200 and 400 mg/kg), and HSP (100 mg/kg), BGL dropped significantly (*p* < 0.05) compared to the untreated diabetic control (DM). In a similar fashion, the levels after day 14 of treatment followed the same trend but with a very significant (*p* < 0.001) reduction in BGL. Treatment with TME (200 and 400 mg/kg) ((DM + TME 200) and (DM + TME 400) groups) reduced BGL more than the HSP group (DM + HSP) (Figure 2). GB attenuated the increase in BGL after induction of diabetes when compared with the DM group. Treatment with TME and HSP showed a nonsignificant increase in BGL when compared with the GB-treated group (DM + GB). The results also revealed that 400 mg/kg TME produced a more significant decrease (*p* < 0.001) in BGL compared to both 200 mg/kg TME and 100 mg/kg HSP treatments.

### 2.3. Histopathological Analysis of Pancreatic Tissue

**Figure 3:**
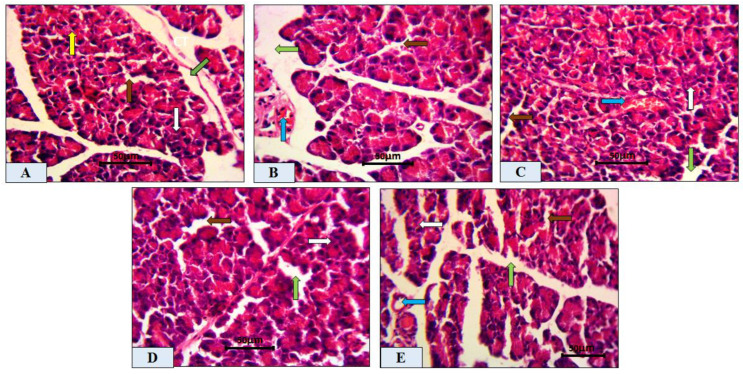
Photomicrographs of control and induced diabetic rat pancreas sections with or without treatment. (**A**) Control rat pancreas (NC) showing normal (exocrine) pancreatic acinar lobules (yellow arrow) and islets of Langerhans (white arrow) embedded within the exocrine portions with vacuolation (brown arrow) and normal intralobular ducts (green arrow). (**B**) Induced diabetic (DM) rat pancreas revealing pathological changes in exocrine acinar lobules with a marked decrease in islets of Langerhans and less apparent cellularity with distorted vacuolation (brown arrow) and blood vessels (blue arrow). (**C**) DM + GB pancreas showing closely packed lobules with improved islets of Langerhans (white arrow), as well as normal interlobular septum ducts (green arrow) and blood vessels (blue arrow), with brown arrows showing normal cellular vacuolation. (**D**,**E**) Induced diabetic rat pancreas treated with DM + TME 400 mg/kg or DM + 100 mg/kg HSP showing improved endocrine cells (white arrow) of the islets of Langerhans, normal vacuolation (brown arrow), improved intralobular septum ducts (green arrow), and normal blood vessels (blue arrow), compared with the DM group. Scale bar = 50 μm. NC, normal control; TME, total methanol extract; HSP, hesperidin; GB, glibenclamide; DM, diabetes mellitus.

The NC group (Figure 3A) showed normal pancreatic lobules consisting of the exocrine portion and islets of Langerhans cells, appearing closely packed with secretory acinar cells, representing the endocrine portion (yellow and white arrows). The acinar cells exhibited intense basal basophilic and apical acidophilic portions. The intralobular duct separating the lobules appeared normal (brown arrow). Figure 3B shows that the DM group exhibited a disturbed lobular architecture with dilated intralobular blood vessels (blue arrow). It showed a marked decrease in the islets of Langerhans (white arrow) with less apparent cellularity compared to the NC group, as well as vacuolation (brown arrow). Islets of DM rats treated with GB Figure 3C showed ameliorated cells with normal pancreatic lobules of both exocrine (white arrow) and endocrine cells, which started to retain their cellularity with normal blood vessels (blue arrow), intralobular ducts (green arrow), and vacuolation (brown arrow). Figure 3D,E show the results for DM rats treated with 400 mg/kg TME and 100 mg/kg HSP, revealing apparently normal pancreatic tissue including the islets of Langerhans (white arrow) and normal vacuolation (green arrow), with blood vessels having a normal architecture (blue arrow), but the amelioration was more prominent in the 100 mg/kg HSP-treated group.

### 2.4. Morphometric Analysis of Pancreatic Tissue

The mean areas of degeneration, inflammatory cell infiltration, and hemorrhage in the untreated diabetic group showed a highly significant (*p* < 0.05) increase in all parameters, while the DM + GB treatment group showed a significant (*p* < 0.05) decrease in all parameters when compared with the untreated diabetic group. No significance difference was observed between DM + GB and DM + HSP groups; while no significant difference was observed between DM + TME and DM + GB groups with respect to degeneration, necrosis, and hemorrhagic parameters (Table 1).

### 2.5. Effect of TME and HSP on Lipid Profile in HFD-Fed STZ-Induced Diabetic Rats

**Figure 4:**
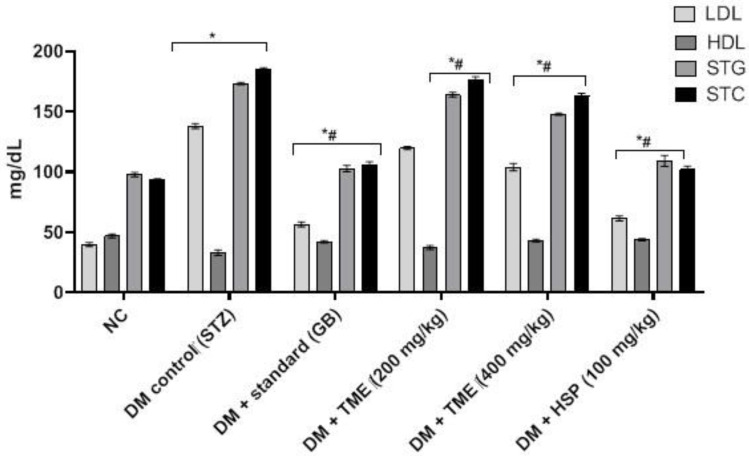
Lipid profile of HFD-fed STZ-induced diabetic rats treated with TME (200 and 400 mg/kg) or HSP (100 mg/kg). Values are represented as mean ± SD (*n* = 6); * significant difference between control group and all other treatment groups; # significant difference between diabetic nontreated group and all treatment groups (TME and HSP). NC, normal control; TME, total methanol extract; HSP, hesperidin; STZ, streptozotocin; GB, glibenclamide; DM, diabetes mellitus; STC, serum total cholesterol; STG, serum triglyceride; LDL, low-density lipoprotein; HDL, high-density lipoprotein.

The levels of serum total cholesterol (STC), serum triglyceride (STG), and low-density lipoprotein (LDL) were significantly (*p* < 0.001) increased in diabetic control rats when compared with the normal control group. However, the high-density lipoprotein (HDL) level in the diabetic control group was lower compared with the normal control group. TME (200 and 400 mg/kg) significantly (*p* < 0.001) reduced the levels of STC, STG, and LDL and significantly (*p* < 0.01) improved the level of HDL compared to diabetic control rats. In contrast, the HSP group more prominently reduced (*p* < 0.001) STC, STG, and LDL levels in addition to significantly improving HDL levels, as shown in Figure 4.

### 2.6. Histopathological Analysis of Adipose Tissue

**Figure 5:**
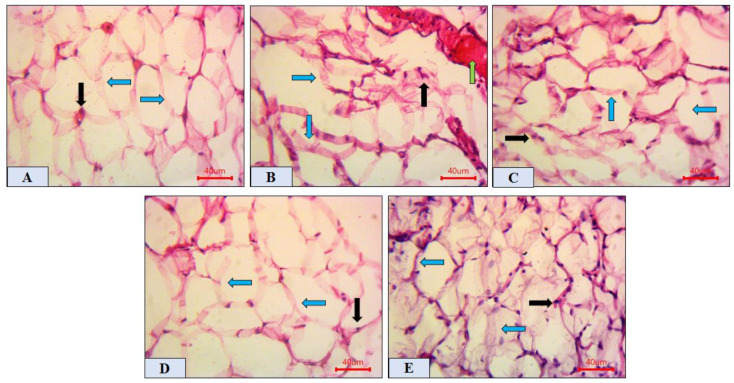
Photomicrographs of rat adipose tissue. (**A**) NC group showing normal polygonal fat cells (blue arrows) and normal adipose nuclei (black arrow). (**B**) Diabetic group showing large-sized, distorted adipocytes (blue arrows) with dilated, congested blood vessels (green arrows) and displaced adipose nuclei (black arrow). (**C**) DM + GB group showing improved adipose tissue (blue arrow) and normalized adipose nuclei (black arrow). (**D**,**E**) DM + TME (400 mg/kg) and DM + HSP (100 mg/kg) group showing apparently normal adipose tissue (blue arrows) and normalized adipose nuclei (black arrow), with a more prominent improvement in DM + TME. Scale bar = 40 μm. NC, normal control; TME, total methanol extract; HSP, hesperidin; GB, glibenclamide; DM, diabetes mellitus.

Adipose tissue photomicrographs of sections from NC, DM, DM + GB, DM + TME, and DM + HSP groups are presented in Figure 5. The NC rats (Figure 5A) showed normal adipose nuclei (black arrow) and normal polygonal adipocytes (blue arrow). Figure 5B shows that the DM group had distorted adipocytes (blue arrow) with intralobular dilated, congested blood vessels and infiltrated leucocytes (green arrow). Figure 5C shows that the DM + GB group displayed improved adipose tissue with normal architecture. The DM + TME group with 400 mg/kg TME (Figure 5D) revealed ameliorated adipocyte tissues (blue arrow) with normalized adipose nuclei (black arrow). Adipose tissue from the DM + HSP group with 100 mg/kg HSP showed apparently normal adipocytes (blue arrow) and normalized adipose nuclei (black arrow), whereas adipocytes of the DM + TME group with 400 mg/kg TME showed better protection, with more polygonal architectural adipocytes compared to the DM + HSP-treated group (Figure 5E).

### 2.7. Effect of TME and HSP on Expression of NF-κB, CASPASE-3, BAX, and BCL2 Markers in Rat Pancreatic Tissue

**Figure 6:**
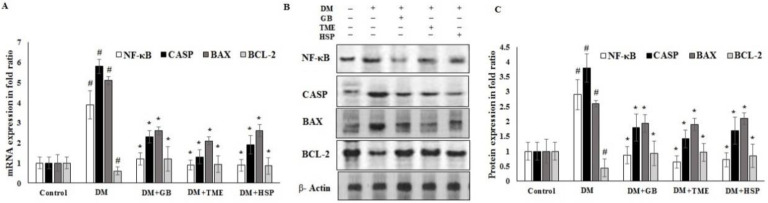
(**A**) Effects of TME, HSP, and GB on the mRNA and protein expression of NF-κB, cleaved caspase-3, BAX, and BCL2 markers of rat pancreatic tissue. The effects of TME, HSP, and GB on NF-κB, cleaved caspase-3, BAX, and BCL2 markers of rat pancreatic tissue were evaluated by real-time PCR. The mRNA of NF-κB, cleaved caspase-3, BAX, and BCL2 markers were quantified using quantitative real-time PCR. GAPDH was used as an internal mRNA control. (**B**,**C**) Alterations in the status of protein expression in response to TME, HSP, and GB were inspected using Western blot. β-Actin was utilized as a control. The experimental data are shown as mean ± SD of triplicate values; * significant difference between DM groups. # significant difference between DM group and control. IM, induction medium; TME, total methanol extract; HSP, hesperidin; GB, glibenclamide; DM, diabetes mellitus; NF-κB, nuclear factor kappa-light-chain-enhancer of activated B cells; CASP, cleaved cysteine aspartic protease-3; BAX, B-cell lymphoma 2 associated X; BCL2, B-cell lymphoma 2.

The results demonstrate that TME and HSP significantly downregulated the mRNA expression of apoptosis markers nuclear factor kappa-light-chain-enhancer of activated B cells (NF-κB), cleaved cysteine aspartic protease-3 (cleaved caspase-3), and B-cell lymphoma 2 associated X (BAX). Similarly, protein expression was significantly downregulated in HFD/DM pancreatic tissue (Figure 6A–C), whereas TME and HSP significantly upregulated the mRNA and protein expression of B-cell lymphoma 2 (BCL2). Furthermore, GB significantly and positively modulated the mRNA and protein expression of all markers in HFD/DM pancreatic tissue compared to the HFD/DM group (Figure 6A–C).

### 2.8. In Silico Binding of HSP and GB with ABC Transporter SUR1

**Figure 7:**
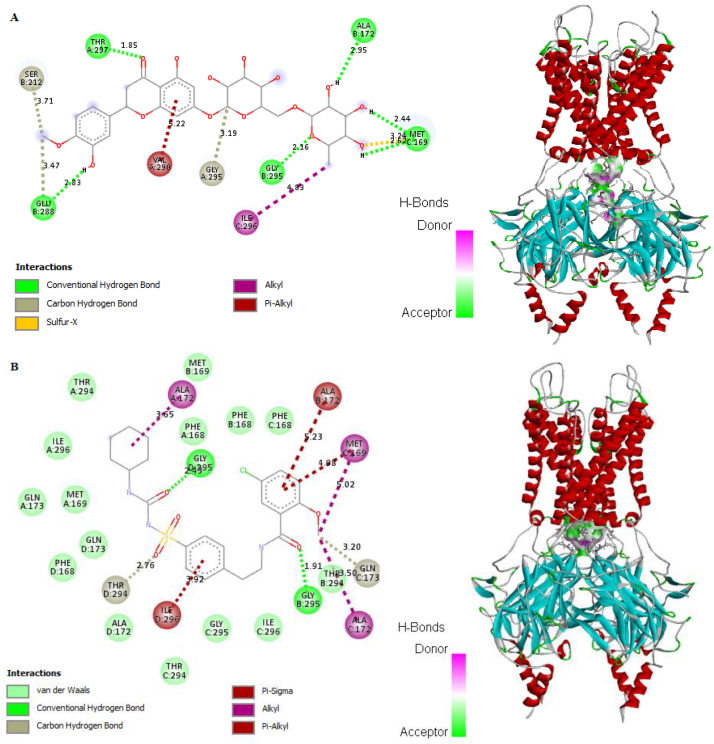
In silico docking and binding interactions of (**A**) HSP and (**B**) GB with ABC transporter SUR1 showing the analysis of amino-acid interactions and their length, together with the binding pocket of ligand–receptor interactions. HSP, hesperidin; GB, glibenclamide.

HSP showed potent binding affinity with ABC transporter SUR1 (−6.18 kcal/mol) of the pancreatic KATP channel protein in comparison to the standard GB (−6.02 kcal/mol). The receptor showed stable binding with ligands (HSP and GB) through formation of hydrogen bonds. Interestingly, HSP formed six hydrogen bonds, four hydrophobic interactions, and one noncovalent sulfur interaction with the receptor protein (Figure 7A and Supplementary S2). On the other hand, GB formed five hydrogen bonds, six hydrophobic interactions, and one pi–pi T-shaped aromatic–aromatic interaction (Figure 7B and Supplementary S2). The greatest energy reduction took place via hydrogen bonds, thus improving the binding strength between the ligands and receptor. These findings revealed that HSP is a highly competitive agonist of the SU receptor and can be considered a suitable analogue of GB.

### 2.9. In Silico Binding of HSP and GB with NF-κB, Cleaved caspase-3, BAX, and BCL2 Markers

**Figure 8:**
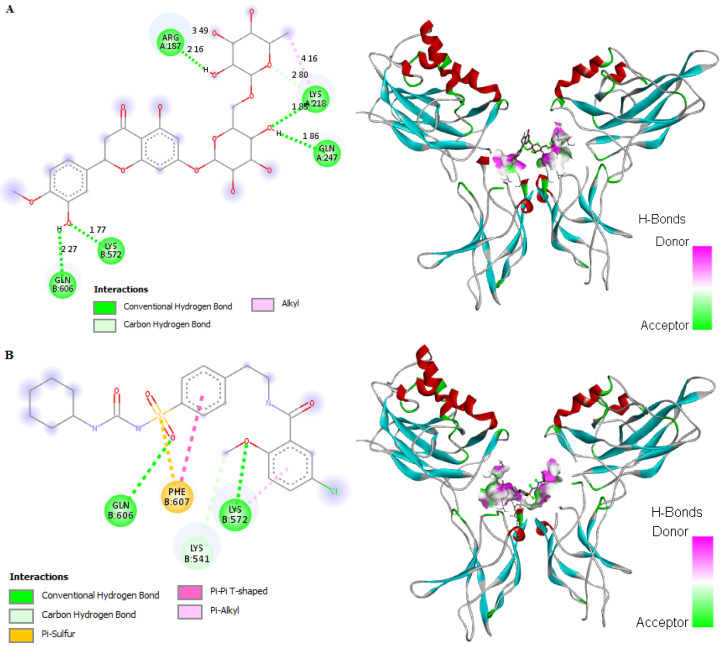

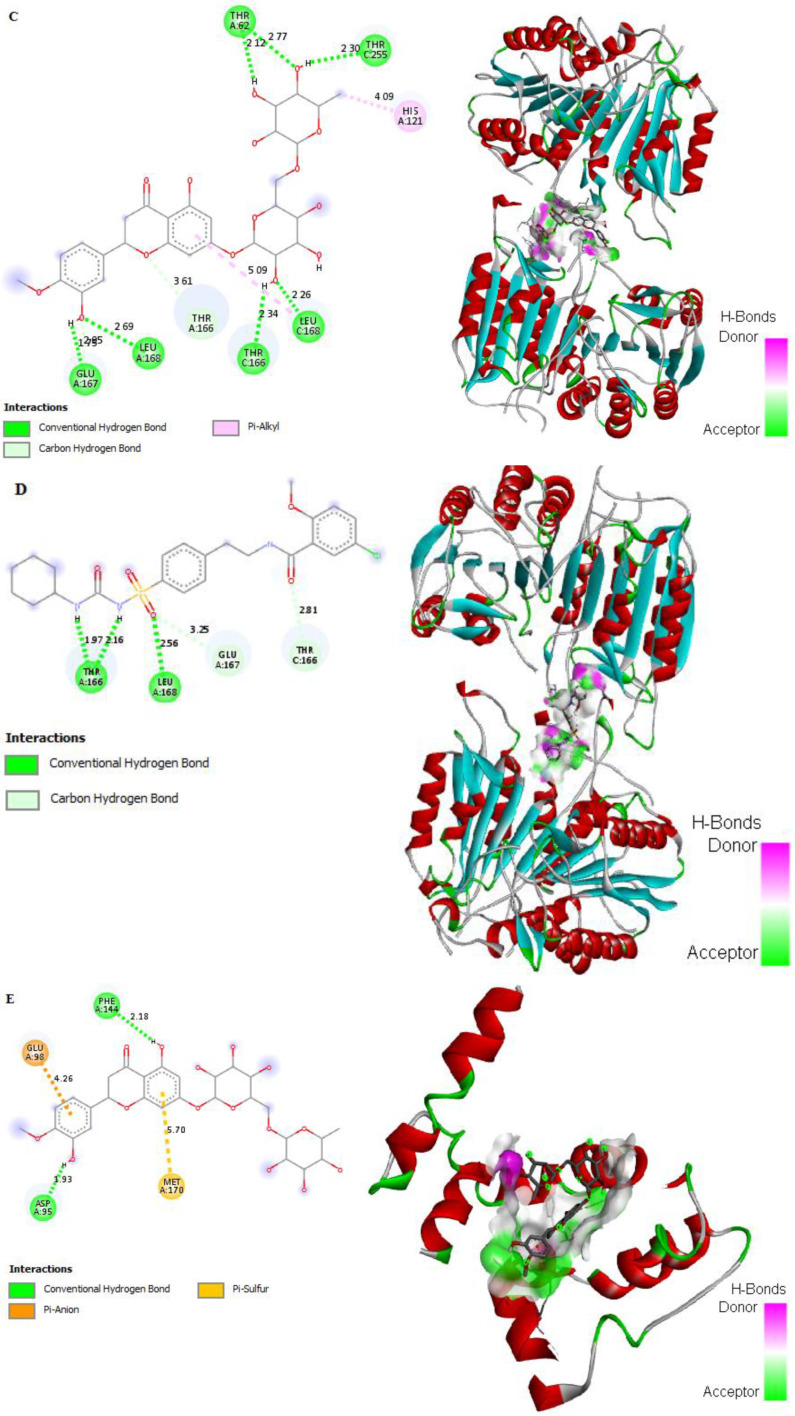

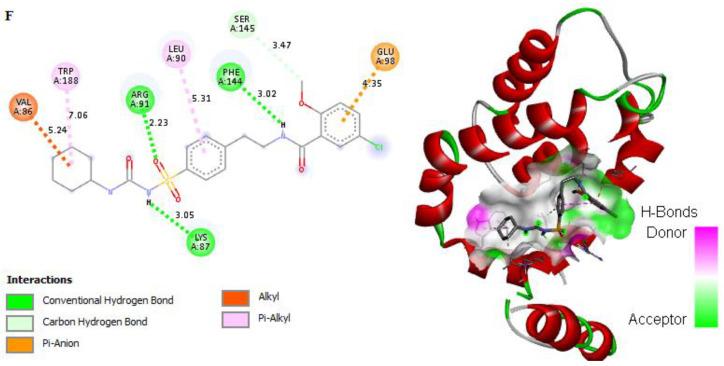
In silico docking and binding interactions of HSP and GB with NF-κB, cleaved caspase-3, and BCL2 markers showing the analysis of amino-acid interactions and their length, together with the binding pocket of ligand–receptor interactions for (**A**) HSP and NF-κB, (**B**) GB and NF-κB, (**C**) HSP and cleaved caspase-3, (**D**) GB and cleaved caspase-3, (**E**) HSP and BCL2, and (**F**) GB and BCL2. HSP, hesperidin; GB, glibenclamide; NF-κB, nuclear factor kappa-light-chain-enhancer of activated B cells; cleaved caspase-3, cysteine aspartic protease-3; BCL2, B-cell lymphoma 2.

HSP showed improved binding affinity with NF-κB and cleaved caspase-3 (−4.92 and −8.44 kcal/mol, respectively) in comparison to the standard GB (−4.70 and −6.35 kcal/mol, respectively). The receptor showed stable binding with both ligands (HSP and GB) through the formation of conventional hydrogen bonds, carbon hydrogen bonds, alkyl hydrophobic interactions, and various pi–alkyl interactions. Interestingly, HSP formed five hydrogen bonds, two carbon hydrogen bonds, and one alkyl hydrophobic interaction with NF-κB amino acids (Figure 8A and Supplementary S2). On the other hand, GB formed three hydrogen bonds, one hydrophobic interaction, one pi–pi T-shaped aromatic–aromatic interaction, and one pi–sulfur interaction (Figure 8B and Supplementary S2). Regarding cleaved caspase-3, HSP showed eight hydrogen interactions and one pi–alkyl hydrophobic interaction (Figure 8C and Supplementary S2), whereas GB showed five hydrogen bonds (Figure 8D and Supplementary S2). Concerning BCL2, HSP and GB demonstrated binding affinities of −6.07 and −6.67 kcal/mol, respectively. HSP formed two hydrogen bonds, one pi–anion interaction, and one pi–sulfur interaction (Figure 8E and Supplementary S2), while GB formed four hydrogen bonds, three alkyl hydrophobic interactions, and one pi–anion interaction (Figure 8F and Supplementary S2). Neither HSP nor GB interacted with BAX amino acids. Most of the energy reduction occurred due to hydrogen bonds, thus improving the binding strength between the ligands and receptors. These findings revealed that both HSP and GB are highly competitive agonists of NF-κB, cleaved caspase-3, and BCL2.

### 2.10. Effect of HSP on Cell Viability and Glucose Uptake of 3T3-L1 Cell Lines

**Figure 9:**
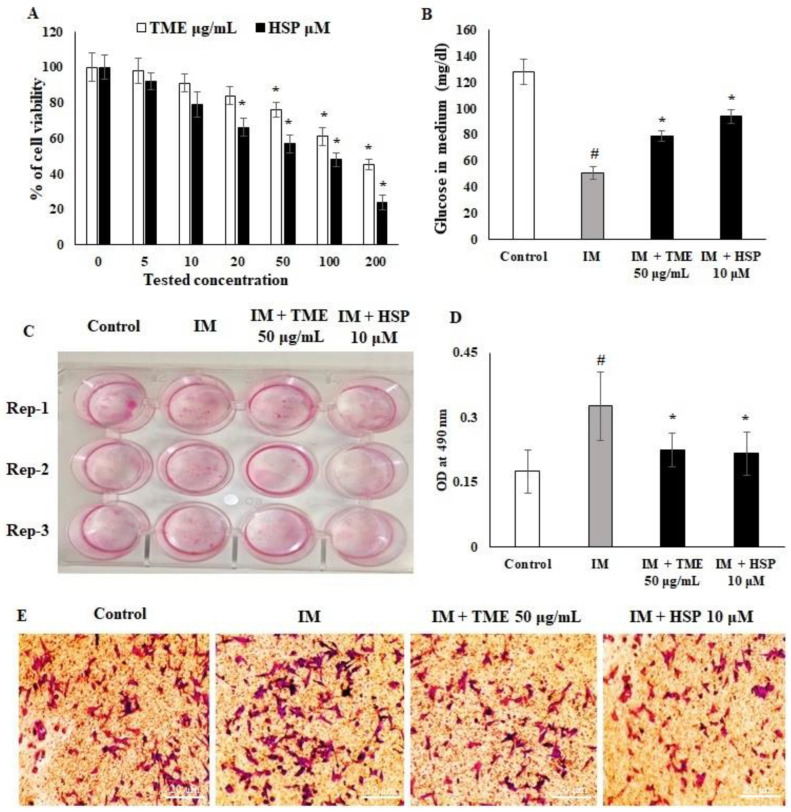
Effect of TME and HSP on 3T3-L1 cell line. (**A**) Cell viability. (**B**) Glucose uptake. (**C**–**E**) Oil Red O staining on day 8 of culture; OD values were measured at 490 nm. The experimental data are shown as mean ± SD of triplicate values; * significant difference from the IM group. # significant difference between IM and control groups. TME; total methanol extract, HSP; hesperidin, IM; induction medium.

The cell viability percentage of 3T3-L1 cells following the administration of TME and HSP was determined, with a 25% reduction observed at 50 μg/mL TME and 10 μM HSP (Figure 9A). The 3T3-L1 cell line was screened in vitro for adipocyte differentiation. Induction medium (IM) containing rosiglitazone is widely applied to induce differentiation in the 3T3-L1 cell line. In this study, medium containing rosiglitazone, insulin, and dexamethasone was applied to induce lipogenesis in the 3T3-L1 cells. The effect of TME and HSP on glucose uptake, intracellular lipid content, and activation of the transcriptional cascade, indicating the mature phenotypic characteristics of adipocytes, was assessed. TME and HSP affected the differentiation of 3T3-L1 cells from pre-adipocytes to adipocytes, with an IC_20_ of 50 μg/mL and 10 μM, respectively. The dose concentration was determined, and the functional concentration was followed for the glucose update assay. The level of glucose in the culture medium was decreased following rosiglitazone-induced adipocyte differentiation. Furthermore, the glucose concentration in the HSP group was increased compared to the induction medium group (Figure 9B). Specifically, it was observed that glucose levels in the medium were increased twofold in response to HSP treatment compared to the IM group. These findings indicate that TME and HSP inhibited glucose uptake from the medium by the 3T3-L1 cell line. Taken together, TME and HSP demonstrated a good reduction in glucose level compared to the control group, suggesting antidiabetic activity, as well as a lower uptake of glucose compared to the IM group, demonstrating a potentially lower lipogenic effect on 3T3-L1 cells.

### 2.11. HSP Inhibited 3T3-L1 Pre-Adipocyte Differentiation to Adipocytes

TME and HSP significantly affected the differentiation of 3T3-L1 cells from pre-adipocytes to adipocytes upon being added to the differentiation medium. On day 8 of culture, as shown by Oil Red O staining, HSP significantly inhibited pre-adipocyte differentiation and the lipid content in intercellular storage. Lipid content was quantified by adding isopropanol to each well to dissolve the Oil Red O, followed by measuring the OD at 490 nm. The results revealed that TME and HSP led to a remarkable decrease in the OD as the concentration increased. Specifically, treatment led to significant decreases in lipid storage content when compared to the IM group (Figure 9C–E).

### 2.12. Effect of TME and HSP on Expression of DGAT1, CEBP/α, and PPARγ in 3T3-L1 Cells

**Figure 10:**
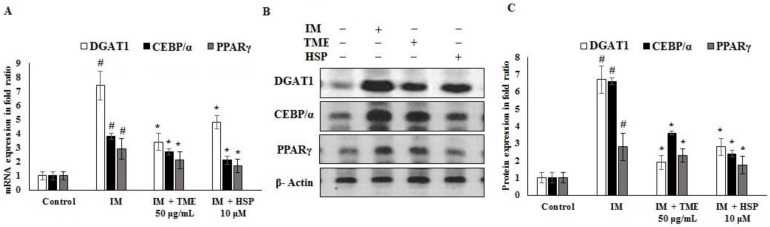
Effects of TME and HSP on mRNA and protein expression of DGAT1, CEBP/α, and PPARγ markers in 3T3-L1 cells evaluated by real-time PCR. 3T3-L1 cells were supplemented with 50 μg/mL TME or 10 μM HSP. (**A**) The mRNA expression of DGAT1, CEBP/α, and PPARγ markers was quantified using quantitative real-time PCR. GAPDH was used as an internal mRNA control. (**B**,**C**) Alterations in protein expression in response to TME and HSP were inspected using Western blot. 3T3-L1 cells were supplemented with 50 μg/mL TME or 10 μM HSP for 24 h. β-Actin was utilized as a control. The experimental data are shown as mean ± SD of triplicate values; * significant difference from IM group. # significant difference between IM and control groups. TME, total methanol extract; HSP, hesperidin; IM, induction medium; DGAT1, diacylglycerol acyltransferase; CEBP/α, CCAAT enhancer-binding protein alpha; PPARγ, peroxisome proliferator-activated receptor gamma.

Transcriptional markers play a major role in differentiation and functional adipogenesis. Diacylglycerol acyltransferase (DGAT1), CCAAT enhancer-binding protein alpha (CEBP/α), and peroxisome proliferator-activated receptor gamma (PPARγ) are receptors that regulate adipogenesis; hence, they were quantified as anti-adipogenesis targets. The results demonstrated that TME and HSP significantly downregulated adipogenesis in the 3T3-L1 cell line (Figure 10A–C). The results showed that mRNA and protein expression of DGAT1, CEBP/α, and PPARγ was upregulated in the IM group, while TME and HSP treatments downregulated their expression. HSP was highly potent in controlling the expression of adipogenic markers in addition to inhibiting lipid storage and glucose uptake in the 3T3-L1 cell line. However, expression of DGAT1 following TME and HSP treatment was more significantly reduced compared to that of CEBP/α and PPARγ (Figure 10A–C).

### 2.13. In Silico Binding of HSP to DGAT1, CEBP/α, and PPARγ Adipogenic Markers in 3T3-L1 Cells

**Figure 11:**
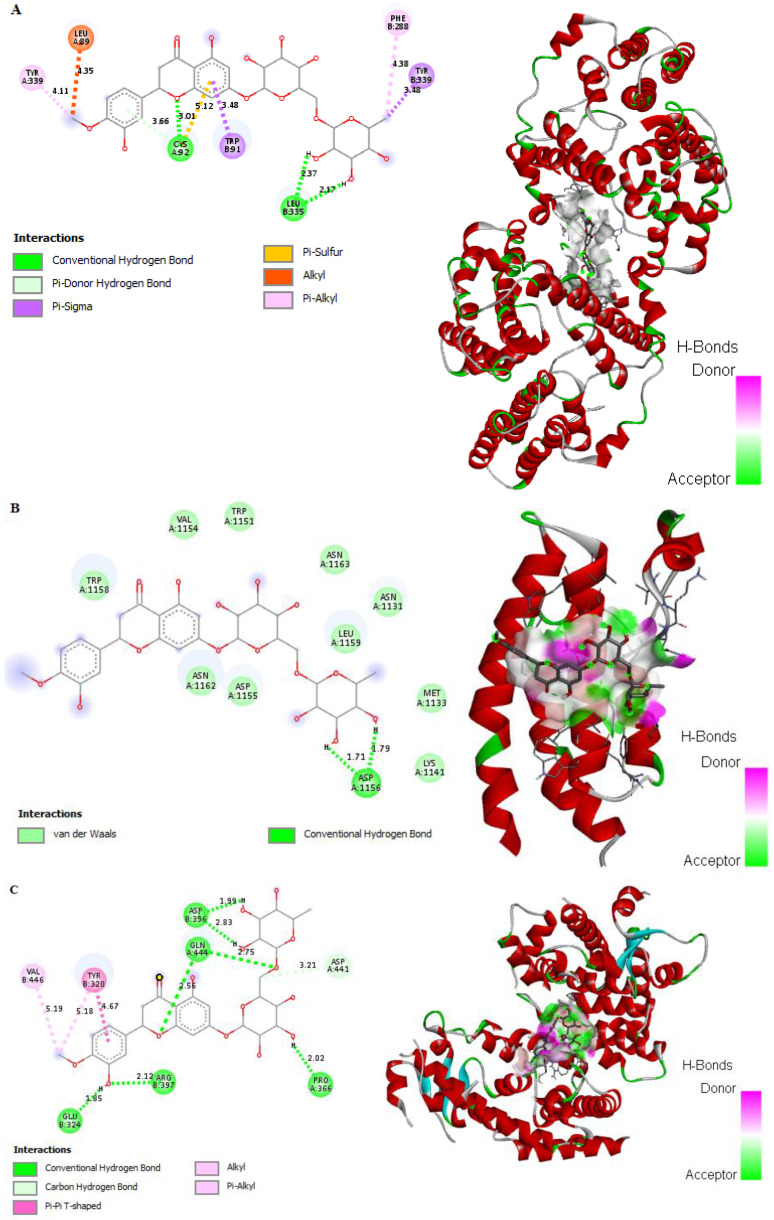
In silico docking and binding interactions of HSP with (**A**) DGAT1, (**B**) CEBP/α, and (**C**) PPARγ receptors, showing the analysis of amino-acid interactions and their length, together with the binding pocket of ligand–receptor interactions. HSP, hesperidin; DGAT1, diacylglycerol acyltransferase; CEBP/α, CCAAT enhancer-binding protein alpha; PPARγ, peroxisome proliferator-activated receptor gamma.

The in silico binding of HSP to DGAT1, CEBP/α, and PPARγ receptors was evaluated to determine its effect on the regulatory proteins. The interaction of HSP with the DGAT1 receptor exhibited a binding energy of −7.07 kcal/mol and intermolecular energy of −8.46 kcal/mol through the formation of nine interactions with cysteine, leucine, tryptophan, and tyrosine of DGAT1 (Figure 11A and Supplementary S2). HSP also interacted with CEBP/α with a binding energy −6.64 kcal/mol and intermolecular energy of −7.54 kcal/mol through the formation of hydrogen bonds with arginine, asparagine, and glutamine, as well as an alkyl hydrophobic interaction with leucine and valine of CEBP/α (Figure 11B and Supplementary S2). HSP demonstrated a binding energy of −4.61 kcal/mol and intermolecular energy of −8.83 kcal/mol toward PPARγ (Figure 11C and Supplementary S2) through interactions with seven amino acids between residues 320 and 444, including pi–alkyl hydrophobic interactions and a covalent interaction with Tyr-320.

## 3. Discussion

Hyperglycemia and hyperlipidemia are two hallmarks of type 2 diabetes mellitus, mainly due to insulin resistance in tissues such as adipocytes [28]. According to the literature, HFD is reported to induce insulin resistance via lipid accumulation in adipose tissues [29], whereas a low dose of STZ destroys the beta cells in the islets of Langerhans [30]. It has been previously reported that HFD induces oxidative and inflammatory characteristics that can contribute to insulin resistance [31]. Therefore, the combination of HFD and low doses of STZ has the potential to precipitate hyperglycemia due to insulin resistance and the elevation of lipid levels [32].

*Ocimum* is considered one of the venerable medicinal plant species of the family Lamiaceae. Various species of *Ocimum* create a wide array of natural products, such as flavonoids, with great medicinal importance, including antidiabetic activity [33,34,35]. *O. forskolei* has been proven to possess several biological activities as shown previously in our in vitro antidiabetic assessment of *O. forskolei* [36]. Hence, the current study was conducted as a thorough assessment of the possible antidiabetic activity of *O. forskolei* (TME) and the isolated pure flavonoid hesperidin (HSP). The current study demonstrated the isolation of HSP from TME (Figure 1). Furthermore, we investigated the effects of TME on HFD/STZ-induced diabetic rats. The HFD/STZ-induced diabetic control showed marked hyperglycemia, particularly compared to the normal control. Our observation in this study is consistent with numerous other studies, proving the role of both STZ and HFD in inducing type 2 diabetes [36,37,38]. The results also revealed that TME (200 and 400 mg/kg) significantly lowered BGL (Figure 2) compared to the diabetic control in a dose-dependent manner, in agreement with previous studies [19,39]. This observation clearly indicates that *O. forskolei* TME has the potential to attenuate hyperglycemic effects. In addition, treatment with TME minimized the destruction of pancreatic islets in STZ-induced diabetic rats (Figure 3). This protection from damage could lead to improved release of insulin and, consequently, enhanced glucose uptake by tissues, as evidenced by the subsequent reduction in BGL [19]. HSP isolated from TME also showed a reduction in the BGL of STZ-induced diabetic rats (Figure 2). Studies have demonstrated that HSP plays numerous protective roles against factors that advance the progression of diabetes mellitus [40]. Furthermore, it has been reported that HSP improves insulin sensitivity by inhibiting inflammatory responses. Our result is in total agreement with many reported studies on the antihyperglycemic activity of HSP [41,42,43].

Evidence from other studies indicated that *Ocimum* extract augmented insulin secretion via its positive effects on pancreatic islet cells [44], which is also supported by the findings of this investigation obtained through histopathology and morphometric analysis of pancreatic cells (Figure 3, Table 1). Histopathological examination showed improved structure of islet cells compared to the damaged pancreatic cells observed in the diabetic control group. Results showed that HFD-STZ-induced untreated diabetic rats exhibited significant degeneration of pancreatic cells, along with hemorrhage and inflammatory cell infiltration. The disruption of pancreatic cell architecture observed in this study is similar to other previously reported studies [45]. In addition, morphometric analysis confirmed pancreatic cell restoration and normalized cellular architectural in the TME-treated diabetic group compared to the untreated diabetic group. This study’s findings agree with the report of Almalki et al. [46]. Following TME and HSP treatment, mRNA and protein expression of apoptotic markers NF-κB, cleaved caspase-3, and BAX was downregulated, while that of antiapoptotic marker BCL2 was upregulated in the pancreatic tissue of treated and diseased groups compared to the control group (Figure 6). Therefore, TME and HSP possibly protect pancreatic tissue against HFD/STZ-induced stress via a modulatory effect on NF-κB, cleaved caspase-3, BAX, and BCL2 biomarkers. The current findings are concordant with reports of the ameliorative effect of natural products on such biomarkers in the HFD/STZ-induced diabetic model [47,48,49,50]. Along with diabetes, hyperlipidemia is a common complication of hyperglycemia [51]. In the present study, we witnessed increased serum levels of STG, STC, and LDL, with a decrease in HDL level in HFD/STZ-induced diabetic control rats. These observations were consistent with the distorted adipocytes, which could be a consequence of fat mobilization according to [18]. However, this study found that TME significantly improved the lipid profile indices of HFD/STZ-induced diabetic rats. The improvement was seen as decreased levels of STC, STG, and LDL, along with enhanced levels of HDL and restoration of the cellular architecture of adipose tissue (Figure 4 and Figure 5). The present results are, therefore, consistent with other documented studies [19,52]. In addition, HSP produced lipid profile characteristics similar to GB in STZ-induced diabetic rats. This finding is consistent with the findings of [41]. The in silico results revealed that both GB and HSP bind to ABC transporter SUR1 via binding to Cys-418. In addition, Cys-418 and Ala-1204 were also involved in alkyl hydrophobic interactions between ABC transporter SUR1 and GB/HSP, revealing the similarity of their binding pockets in SUR1 (Figure 7, Supplementary S2). The ABC transporter has been reported to function through nucleotide-binding domain (NBD) dimerization [53]. Targeting SUR1 using sulfonylureas to inhibit the ATP-sensitive potassium channel protein has been adopted as a strategy to promote insulin secretion [54]. Subsequent structural studies revealed that GB binds to NBD, preventing ATP/ADP-mediated NBD closure and regulating SUR-mediated channel activity [55]. Altogether, the present computational analysis revealed that both GB and HSP have a similar binding pocket in ABC transporter SUR1, which is involved in the regulation of ATP-sensitive potassium channels (Figure 7, Supplementary S2). Similarly, the in silico results revealed that both GB and HSP interacted with NF-κB, cleaved caspase-3, BAX, and BCL2, confirming their protective effect on pancreatic tissues (Figure 8, Supplementary S2).

Increased fat mass and body weight are observed in type 2 diabetes, leading to a higher amount of lipid storage in adipose tissue and its associated cells [56]. Reducing glucose uptake and fat storage is a significant prognostic approach to controlling type 2 diabetes. Insulin stimulates fibroblastic adipose tissue stores to increase lipid accumulation via upregulation of DGAT1, CEBP/α, and PPARγ [57,58]. In this study, TME and HSP reduced the cell proliferation rate of 3T3-L1 cells to 80% at 50 μg/mL and 10 μM, respectively (Figure 9), confirming the nontoxicity of TME and HSP toward adipose cells at these concentrations. Mechanistically, glucose uptake was significantly inhibited by 50 μg/mL TME and 10 μM HSP (Figure 9). The adipogenic markers DGAT1, CEBP/α, and PPARγ are the key regulatory factors of adipogenesis in liver and adipose cells [59,60]. The results indicated that TME and HSP reduced mRNA and protein expression of DGAT1, CEBP/α, and PPARγ (Figure 10). A significant reduction was observed in all targets, with PPARγ showing the most significant reduction, thus inhibiting lipogenesis and fatty-acid biosynthesis (Figure 10). Moreover, in silico interaction studies confirmed the regulatory effect of HSP on adipogenic markers DGAT1, CEBP/α, and PPARγ (Figure 11 and Supplementary S2). The current findings reveal the antidiabetic and antihyperlipidemic role of TME and HSP in the HFD/STZ-induced diabetic rat model. This study also confirmed their modulatory effect on diabetes-mediated adipogenic factors in 3T3-L1 adipocytes through in silico docking interactions.

## 4. Materials and Methods

### 4.1. General Experimental Procedures and Chemicals

Two-dimensional ^1^H- and ^13^C-NMR spectra were measured on an Avance 400 NMR spectrometer (^1^H-NMR: 400 MHz and ^13^C-NMR: 100 MHz, Bruker, Uster, Switzerland). Diaion HP-20 (Sigma-Aldrich, Darmstadt, Germany) and Sephadex LH-20 (Sigma-Aldrich, St. Louis, MO, USA) columns, along with precoated silica gel 60 F254 plates, 0.25 mm and 1000 μm in thickness (Sigma Aldrich, Darmstadt, Germany), were used for thin-layer chromatography (TLC), applying 10% vanillin in ethanol as the visualizing agent with a hotplate (150 °C). Analytical-grade chemicals and reagents were used. Streptozotocin (STZ) and glibenclamide (GB) were obtained from Sigma-Aldrich (St. Louis, MO, USA).

### 4.2. Plant Material

*O. forskolei* Benth was purchased from a local market, Al-Ahsa region (April 2016). *O. forskolei* was kindly identified by Eng. Mamdouh Shokry, Director of El-Zohria Botanical Garden, Giza, Egypt. A voucher specimen of the plant is deposited in the Herbarium of the Department of Pharmaceutical Sciences, College of Clinical Pharmacy, King Faisal University, Al-Ahsa, Saudi Arabia (16-Apr-OF).

### 4.3. Extraction and Isolation of the Major Plant Constituents

The carefully shade- and air-dried powdered areal parts of *O. forskolei* (10 kg) were exhaustedly extracted by cold maceration using 70% methanol at room temperature. The compiled extracts were then concentrated under reduced pressure, yielding the total methanol crude extract (600 g). The total methanol extract (TME) (500 g) was suspended in deionized water and then partitioned with *n*-hexane to give the hexane fraction (120 g), while the remaining mother liquor was concentrated to give defatted TME (280 g). The defatted TME (28 g) was subjected to Diaion HP-20 CC (4 kg; water (20 L), methanol (20 L), and acetone (8 L)) to give the water-soluble fraction (70 g), methanol-soluble fraction (130 g), and acetone-soluble fraction (80 g), respectively. During concentration of the methanol-soluble fraction (130 g), a heavy precipitate was noted and collected. The precipitate was further purified by Sephadex LH-20 column chromatography, eluted with 50% methanol, and monitored by TLC, yielding hesperidin (HSP)-containing subfractions. HSP-containing subfractions were compiled and concentrated before being subjected to Sephadex LH-20 column chromatography using butanol saturated with water as the mobile phase to finally yield pure hesperidin (HSP) (1.3 g).

### 4.4. Animals

Thirty-six male Wistar rats were obtained from Nahda University, Beni Suef (NUB) Animal Care Facility. The animals were housed under standard laboratory conditions and maintained on a 12-h light/dark cycle to allow acclimatization for 2 weeks. Animals were allowed free access to food and water, following the procedure for animal experiments described by the Minia University Ethical Committee (ES13/2020). Animals were then randomly assigned to six treatment groups consisting of six rats per group.

### 4.5. Diet

Rat diet composition was adopted according to Mirghani et al. [61]. A normal rat chow diet containing 3% fat (soy oil), 18% casein protein, 69% carbohydrates, 9% minerals, and 1% vitamins (per 100 g) was used for the control. The high-fat diet for the diabetic group consisted of 40% fat (20% soy oil and 20% lard fat oil), 14% casein protein, 37% carbohydrates, 8.2% minerals, and 0.8% vitamins.

### 4.6. Design of Experiment

Treatment groups were divided into two main categories: the control group (nondiabetic, normal diet; NC group) and the diabetic group (high-fat diet (HFD) with 40% fat for 2 weeks, followed by a single intraperitoneal injection of STZ dissolved in 0.1 M sodium citrate buffer at pH 4.4, 35 mg/kg, for induction of type 2 diabetes) [62]. The diabetic group (with a fasting blood glucose level >250 mg/dL) was then subdivided into four groups comprising nontreated diabetic rats (DM group), diabetic rats treated with 50 mg/kg GB (DM + GB group), diabetic rats treated with 200 and 400 mg/kg TME suspended in sterile water (DM + TME 200 and DM + TME 400 groups, respectively), and diabetic rats treated with 100 mg/kg HSP (DM + HSP group). Blood samples were collected via the tail vein for measurement of blood glucose levels using glucose–oxidase–peroxidase reactive strips (Accu-Chek Active, Roche Diagnostics GmbH, Mannheim, Germany). Lipid parameters were evaluated using an automated chemistry analyzer (Merck, Wiesbaden, Germany). Treatments were given orally and continued for 2 weeks, after which all rats were sacrificed for tissue collection.

### 4.7. Tissue Collection

Animals were anaesthetized before their pancreas and epididymal fat were dissected out and then perfused with cold saline in 10% neutral buffered formalin overnight, followed by processing to obtain paraffin blocks. After staining with hematoxylin and eosin, serial paraffin sections of 6 μm thickness were cut and prepared for histological examination, as previously described by Abdelwahab et al. (2021) [63]. The slide sections were examined using a light microscope (Olympus, Tokyo, Japan). Photomicrographs were digitally captured using a high-resolution color digital camera (Olympus, Tokyo, Japan) adapted to the microscope and connected to a computer.

### 4.8. Morphometric Analysis

Using image analyzer software (ImageJ v1.47, National Institutes of Health, Bethesda, MD, USA), the percentage area of connective tissue and the intensity of the brown color of anti-insulin immune expression were calculated. Quantitative data were collected for three parameters. Pancreatic histological scoring was performed on H&E-stained slides from each rat at a magnification of ×400. Scoring was carried out on a scale of 0–4 for the parameters of degeneration, lymphocytic infiltration, and hemorrhage by counting the mean number of affected foci as follows: 0 = absent, 1 = mild, 2 = moderate, 3 = severe, and 4 = overwhelming. The degree of all previous parameters were measured semi-quantitatively in 10 random fields within each slide (three sections per animal) [64,65].

### 4.9. Computational Studies

Computational studies were carried out to explain the molecular basis underlying the interactions of HSP (CID-10621) and GB (CID-3488) ligands with the ATP-binding sulfonylurea receptor. The crystal structures of the ABC transporter (ATP-binding cassette of the sulfonylurea receptor) of pancreatic KATP channel protein (SUR1) (PDB ID: 6c3o; Chain E), DGAT1 (PDB ID: 6vz1), CEBP/α (PDB ID: 1nwq), PPARγ (PDB ID: 3et0), BCL2 (PDB ID: 5c3g), BAX (PDB ID: 5w62), cleaved caspase-3 (PDB ID: 3dek), and NF-κB (PDB ID: 1vkx) were retrieved from the Protein Data Bank (www.rcsb.org, accessed on 10 February 2022). The protein structure was prepared and optimized by the protein preparation modules in the assorted tools of the WhatIF server, PyMol, and AutoDock software package, as described earlier by [66]. The water molecules and crystal-bound molecules in the receptors were removed. The protein structures were protonated and optimized in physiological pH conditions by adding polar molecules. The AutoDock grid was centered around the co-crystallized ligand in a box size of 15 Å. Then, the docking module was used for docking the compounds into the SU receptor. The docking scores were calculated using AutoDock analysis.

### 4.10. Cell Culture

The 3T3-L1 cell line was procured from KFSHRC, Saudi Arabia, and maintained by the Molecular Biology Laboratory, College of Science, King Faisal University, Saudi Arabia. Cells were cultured in Dulbecco’s modified Eagle’s medium (DMEM) containing 10% FBS and 25 mM HEPES (Sigma, St Louis, MO, USA) at 37 °C with 5% CO_2_. Differentiation of adipocytes was achieved by induction with 10 μg/mL insulin (Sigma, St Louis, MO, USA), 1 μM dexamethasone, and 10 μM rosiglitazone (Sigma, St Louis, MO, USA) differentiation medium (DM), with TME and HSP added for 7 days. During this process, the differentiation medium was replenished every 36 h [67]. On day 8 of culture, all groups of 3T3-L1 cells were analyzed for Oil Red O staining, glucose uptake, and mRNA and protein expression of adipogenic markers.

### 4.11. Measurement of Glucose Content in Medium

The 3T3-L1 cells were cultured in 48-well plates up to differentiation. The cell-free supernatants were collected, and total glucose was quantified using BioTek microplate reader (BioTek, Winooski, VT, USA) (Cayman chemicals, Ann Arbor, MO, USA) according to the manufacturer’s protocols, with values expressed as mg/dL.

### 4.12. Oil Red O Staining

After day 7 of 3T3-L1 cell differentiation, cells treated or not treated with HSP were washed with PBS and fixed with 4% paraformaldehyde for 30 min, then stained with Oil Red O [68] (Sigma, St Louis, MO, USA) at 4 °C for 60 min. Following staining, the cells were aspirated twice with PBS and fixed using 8% methanol. The lipid content in the cells was photographed using an XLcore Life Technologies microscope at 200× magnification with EVOS XL core imaging (Life Technologies, Austin, TX, USA). In brief, cell supernatants were removed, and the lipid-loaded Oil Red O stain was dissolved using isopropanol. Then, 200 μL aliquots from each well were transferred to a new 96-well plate, and the OD value was measured at 490 nm using a BioTek ELISA reader (BioTek, Winooski, VT, USA).

### 4.13. mRNA Quantification

The TME- and HSP-treated 3T3-L1 cells were cultured for 8 days in a CO_2_ incubator. The treated 3T3-L1 cells and collected rat pancreatic tissues were washed with ice-cold PBS, and the cell-free supernatant was removed. Total RNA was extracted using the modified Trizol (Thermo Fisher, San Jose, CA, USA) method. The extracted RNA was quantified using NanoDrop, and 300 ng of mRNA was used for cDNA preparation using the MQ basic cDNA synthesis kit (Molequle-on, Takara, Kusatsu, Shiga, Japan) [69]. The cDNA was amplified according to the primers summarized in Table 2, and mRNA expression was quantified using ΔΔCt values.

### 4.14. Western Blot Analysis

TME- and HSP-treated 3T3-L1 cells were collected after 7 days of treatment and trypsinized using ice-cold PBS. The cell pellet was collected and lysed using Santa Cruz RIPA lysis buffer (Santa Cruz, Paso Robles, CA, USA). The extracted proteins were quantified, and 50 µg of protein was loaded in SDS-PAGE. Similarly, for pancreatic tissues, pancreatic homogenate was prepared, and 45 μg of pancreatic protein was used. The separated protein was transferred to polyvinylidene difluoride (PVDF) membranes (pore size: 0.45 µm, Bio-Rad, Hercules, CA, USA). Transferred blots were probed with primary antibodies overnight at 4 °C according to the manufacturer’s protocol. The primary antibodies CEBP/α (mouse monoclonal antibody, 1:1000; Biorybt, Cambridge, UK), PPARγ (rabbit polyclonal antibody, 1:2000; Biorybt, Cambridge, UK), BAX (rabbit polyclonal antibody, 1:750; Biorybt, Cambridge, UK), BCL2 (rabbit polyclonal antibody, 1:1000; Biorybt, Cambridge, UK), cleaved caspase-3 (rabbit polyclonal antibody, 1:1000; Cell Signaling, Danver, MA, USA), DGAT (rabbit polyclonal antibody, 1:1500; Biorybt, Cambridge, UK), NF-κB-p65 (rabbit polyclonal antibody, 1:2000; Thermo Fisher Scientific, Waltham, MA, USA), and β-actin (rabbit polyclonal antibody, 1:2000; Cell Signaling Technology, Beverly, MA, USA) were incubated overnight at 4 °C and then washed with TBST. Washed blots were incubated with horseradish peroxidase-conjugated secondary antibody at room temperature for 1 h. The blots were visualized by an enhanced chemiluminescence (ECL) system (Pierce, Life Technologies, Austin, TX, USA) and scanned using a LICOR detection system. Expressed bands were analyzed using ImageQuant software and quantified by densitometry using ImageJ software v1.8 [70].

### 4.15. Data Analysis

Data analysis was performed using SPSS version 20 (SPSS Inc., Chicago, IL, USA) and GraphPad Prism software version 8.2 (San Diego, CA, USA). Data were expressed as mean ± standard deviation (mean ± SD). Significant differences between groups were carried out by two-way ANOVA, and Tukey’s multiple comparison test was used to compare between groups. A *p*-value < 0.05 was considered statistically significant.

## 5. Conclusions

The current study revealed the antidiabetic and anti-obesity properties of *Ocimum forskolei* for the first time. The study demonstrated the potential of the TME of *Ocimum forskolei* and its constituent HSP to control BGL and serum lipid levels in HFD/STZ-induced diabetic rats. In addition, the results suggested the ability of the TME of *Ocimum forskolei* and HSP to restore normal pancreatic and adipose tissue architecture. The TME of *Ocimum forskolei* and HSP demonstrated the regulation of apoptotic markers NF-κB, cleaved caspase-3, BAX, and BCL2 in pancreatic tissue. On the other hand, TME and HSP reduced the uptake of glucose and the oxidative lipid accumulation in 3T3-L1 cells via modulation of mRNA and protein expression of DGAT1, CEBP/α, and PPARγ. Furthermore, virtual binding studies supported the results, revealing potent binding of HSP with ABC transporter SUR1, DGAT1, CEBP/α, PPARγ, NF-κB, cleaved caspase-3, BAX, and BCL2. These findings highlight the potential of the TME of *Ocimum forskolei* and HSP as beneficial therapeutic agents to treat elevated blood glucose levels and other lipid profile biomarkers in diabetes and obesity-related conditions. Future clinical pharmacokinetic trials on the TME of *Ocimum forskolei* and HSP are recommended to validate their approval as potential antidiabetic and anti-obesity agents.

## Figures and Tables

**Table 1:** Effect of diabetes and different treatments on pancreatic morphology.

Groups	NC	DM	DM + GB	DM + TME	DM + HSP
Degeneration and necrosis	0.14 ± 0.11	6.43 ± 0.37 ^ac^	4.43 ± 0.30 ^ab^	2.86 ± 0.26 ^abc^	4.71 ± 0.42 ^ab^
Mononuclear cellular infiltration	0.63 ± 0.26	23.14 ± 0.74 ^ac^	14.00 ± 1.02 ^ab^	9.29 ± 0.97 ^a^	14.57 ± 1.04 ^ab^
Hemorrhage	1.13 ± 0.25	4.00 ± 0.27 ^ac^	2.75 ± 0.16 ^ab^	1.63 ± 0.26 ^abc^	3.00 ± 0.27 ^ab^

Results are presented as mean ± SD (*n* = 6). ^a^ significant difference from control group; ^b^ significant difference from DM group; ^c^ significant difference from DM + GB group, *p* < 0.05. NC, normal control; TME, total methanol extract; HSP, hesperidin; GB, glibenclamide; DM, diabetes mellitus.

**Table 2:** Real-time PCR primer details.

Primer Name	Forward	Reverse	PCR Product Size in Base Pair (bp)
PPARγ	GAAAGACAACGGACAAATCACC	GGGGGTGATATGTTTGAACTTG,	169
CEBP/α	TTGTTTGGCTTTATCTCGGC,	CCAAGAAGTCGGTGGACAAG	
DGAT1	CTACAGGTCATCTCAGTGCT	GAAGTAGAGCACAGCGATGA	121
BAX	TGGCAGCTGACATGTTTTCTGAC	TCACCCAACCACCCTGGTCTT-	195
BCL2	TCGCCCTGTGGATGACTGA	CAGAGACAGCCAGGAGAAATCA	134
CASPASE-3	GTGGAACTGACGATGATATGGC	CGCAAAGTGACTGGATGAACC	211
NF-κB	CATGAAGAGAAGACACTGACC ATGGAAA	TGGATAGAGGCTAAGTGTAGA CACG	310
GAPDH	CGTCCCGTAGACAAAATGGT,	TTGATGGCAACAATCTCCAC	212

PPARγ, peroxisome proliferator-activated receptor gamma; CEBP/α, CCAAT enhancer-binding protein alpha; DGAT1, diacylglycerol acyltransferase; BAX, BCL2 associated X; BCL2, B-cell lymphoma 2; CASPASE-3, cleaved caspase 3 (cleaved cysteine aspartic protease-3); NF-κB, nuclear factor kappa-light-chain-enhancer of activated B cells; GAPDH, glyceraldehyde 3-phosphate dehydrogenase.

## Data Availability

The data presented in this study are available on request from the corresponding author.

## Supplementary Materials

The following supporting information can be downloaded at: https://www.mdpi.com/article/10.3390/molecules27092800/s1: Supplementary S1. 1D- and 2D-NMR spectroscopic data; Supplementary S2. Interactions of HSP and GB with amino-acid residues of corresponding docked receptors.

## Sample Availability

Sample of hesperidin is available from the corresponding author.
