# Supplementary material for: Ameliorative Effect of Ocimum forskolei Benth on Diabetic, Apoptotic, and Adipogenic Biomarkers of Diabetic Rats and 3T3-L1 Fibroblasts Assisted by In Silico Approach
Source: Molecules. 2022 Apr 28;27(9):2800. doi: 10.3390/molecules27092800 (PMC9101318; doi:10.3390/molecules27092800)
Supplement: Supplementary file 1 [file molecules-27-02800-s001.zip › Supplementary‎-S1‎.pdf]

Supplementary-S1: 1D- and 2D- NMR spectroscopic data of Hesperidin

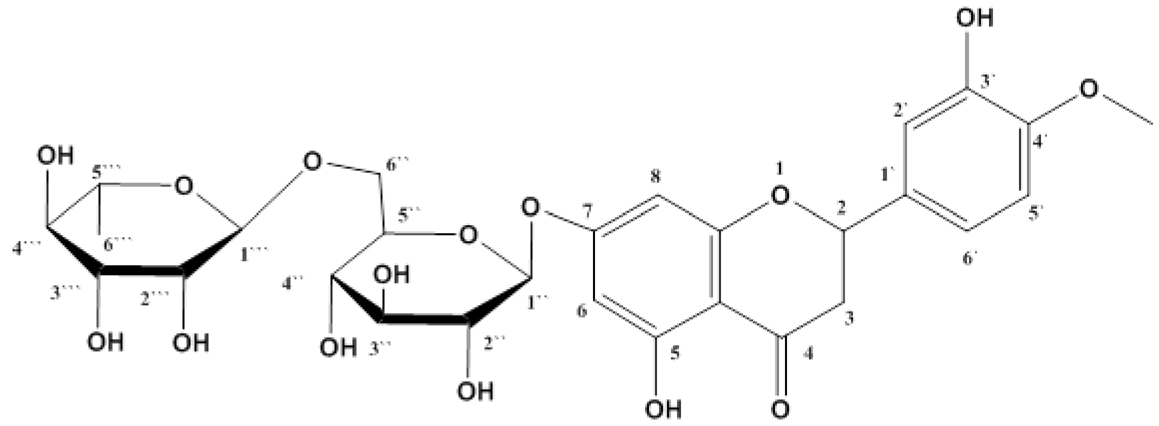

Structure of Hesperidin

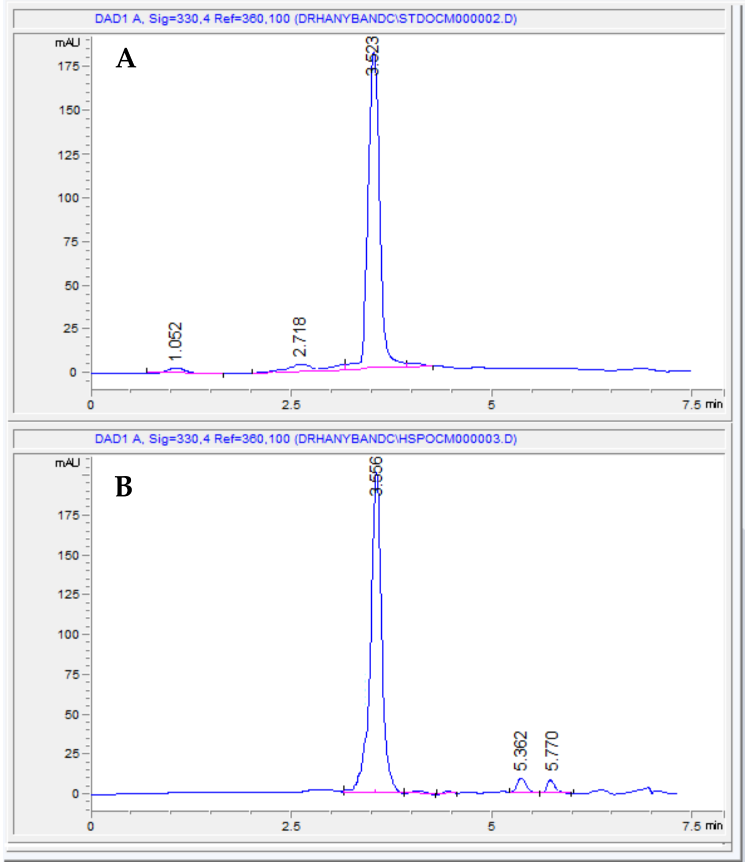

HPLC chromatograms of authentic hesperidin (A) and isolated hesperidin (B)

High performance liquid chromatography (HPLC) apparatus type (Agilent, 1200 series, Germany) equipped with PDA detector and Discovery® C18 (5 Cm x 4.6 mm x 5 µm) column (supelco, USA). An isocratic mode elution equipped with Acetonitrile: water blend (75:25 v/v) with flow rate 1mL/min. injection volume was 10 µL (from 5 mg/mL, samples in methanol(. The chromatogram was monitored using Agilent Chemstation software (Version B.2.4.1)

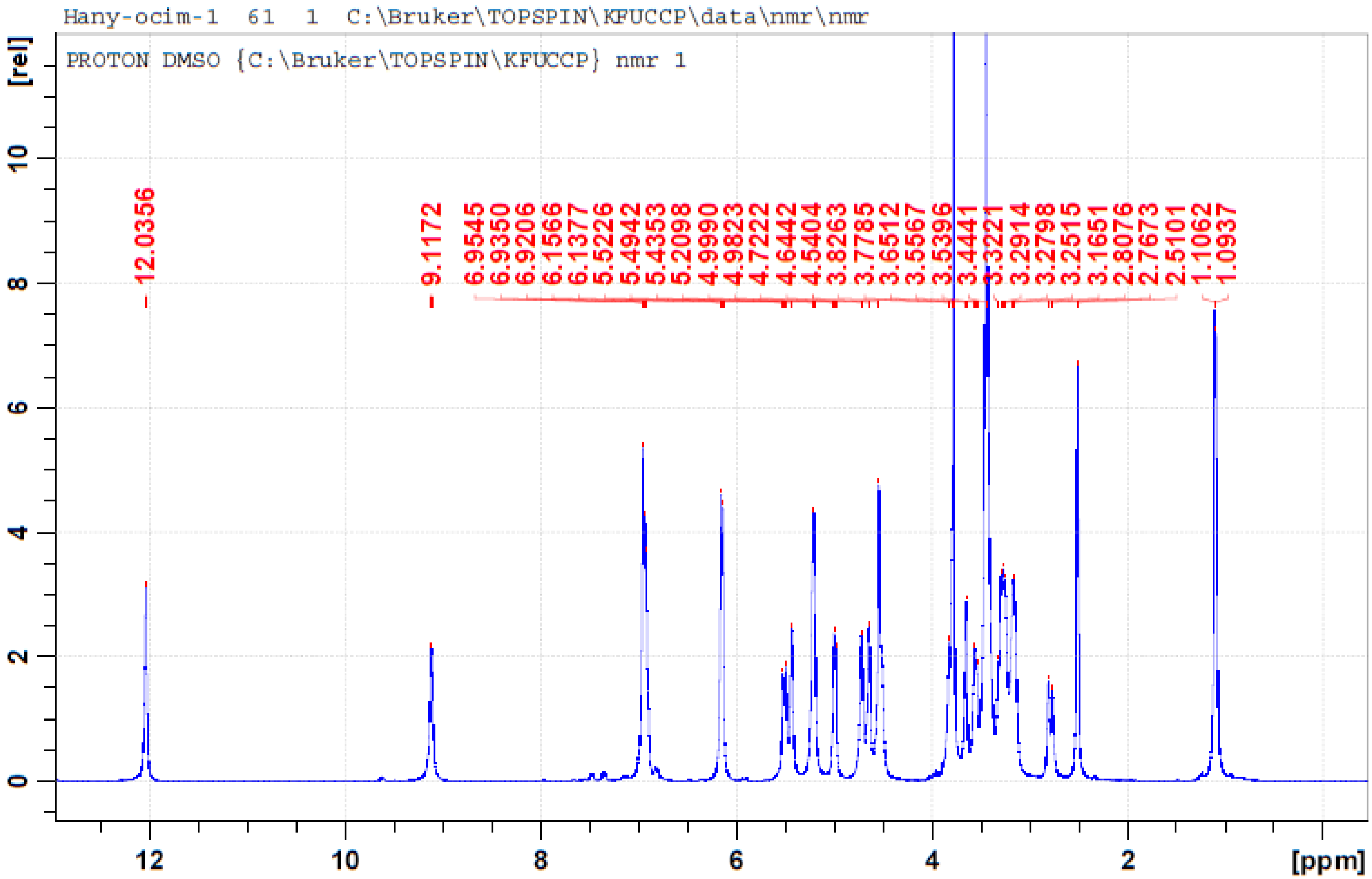

1-^{1}H-NMR spectrum of Hesperidin (400 MHz, DMSO-*d6*)

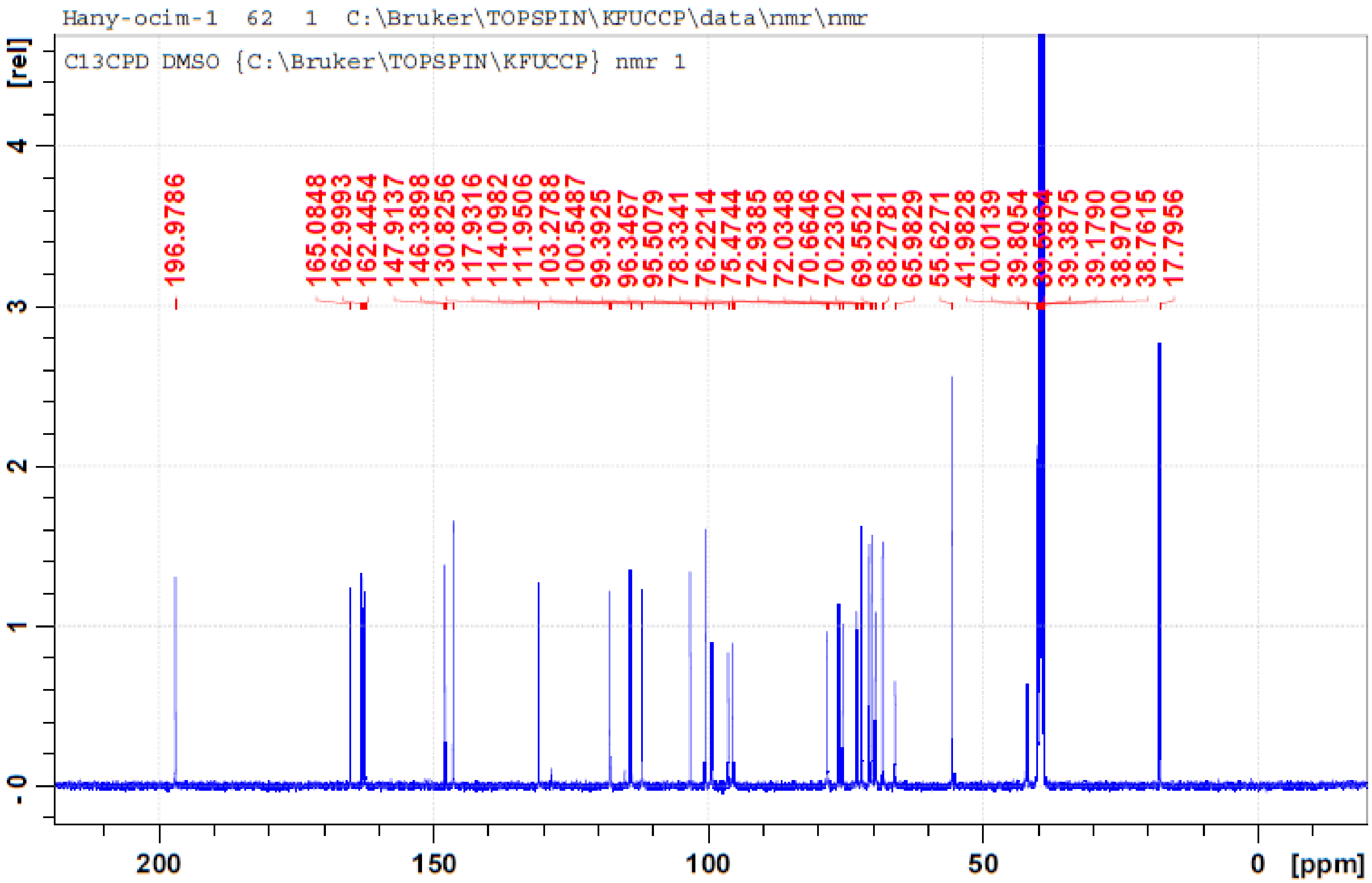

2- ^{13}C-NMR spectrum of Hesperidin (100 MHz, DMSO-*d6*)

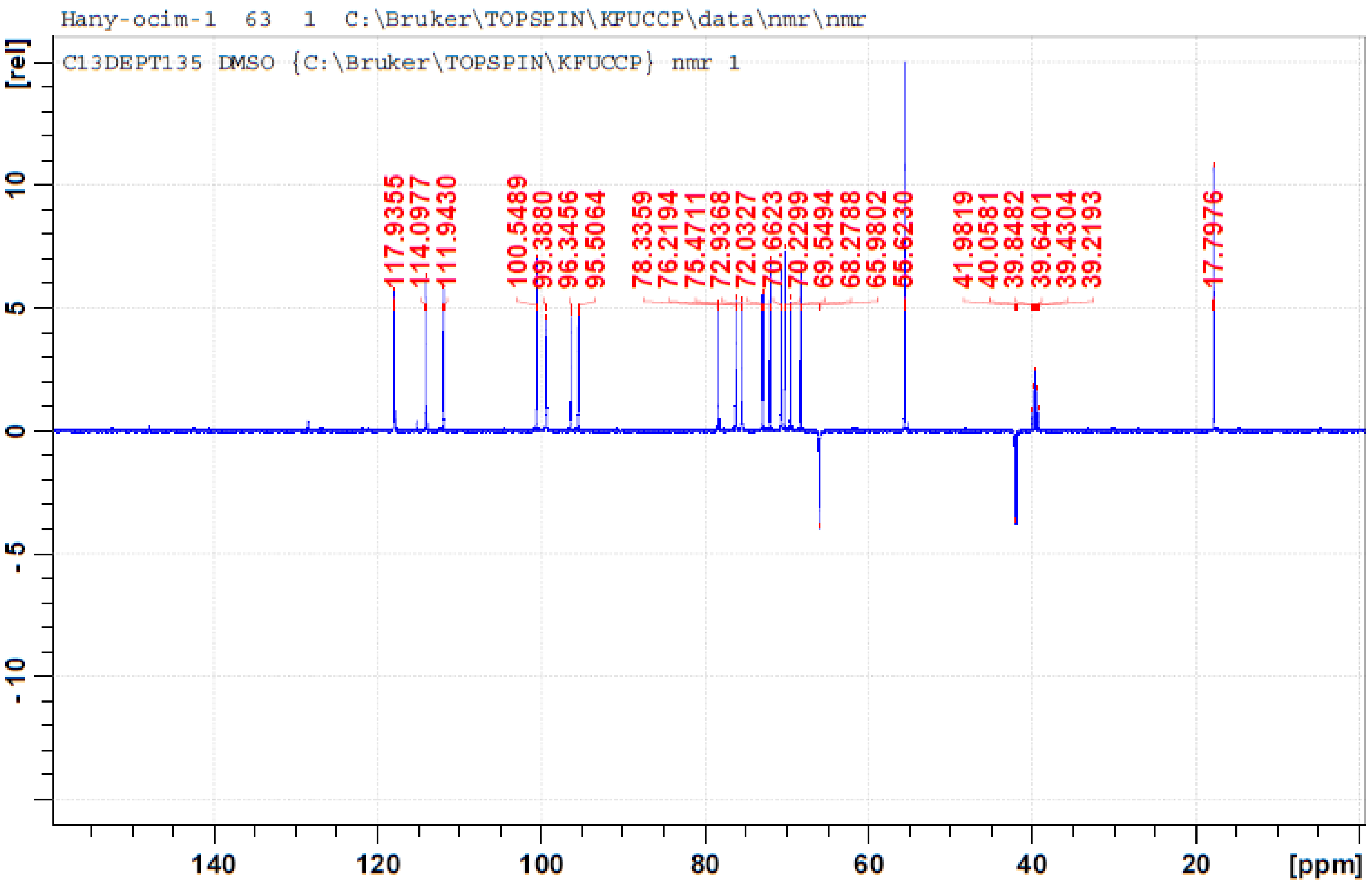

<u>**3- DEPT spectrum of Hesperidin (100 MHz, DMSO-*d6*)**</u>

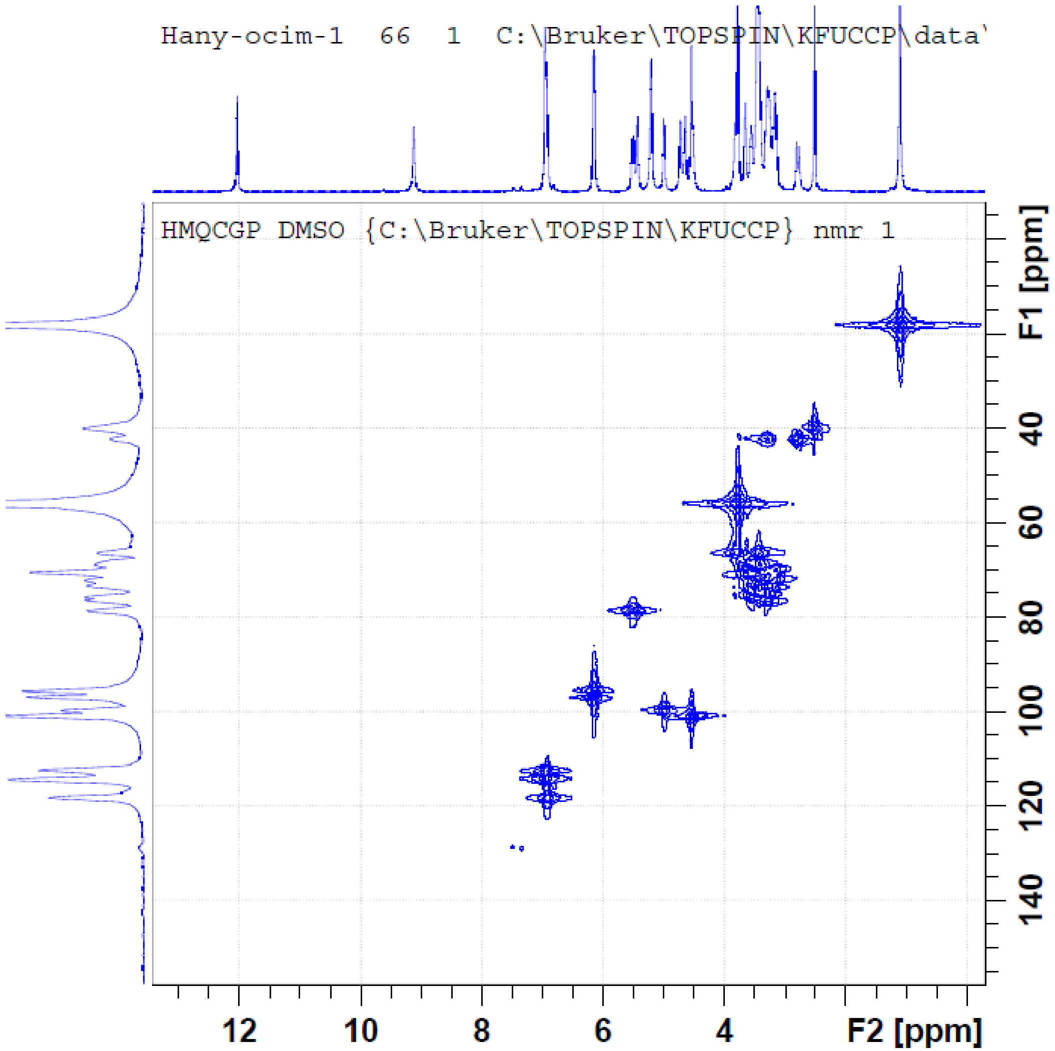

4- HMQC spectrum of Hesperidin

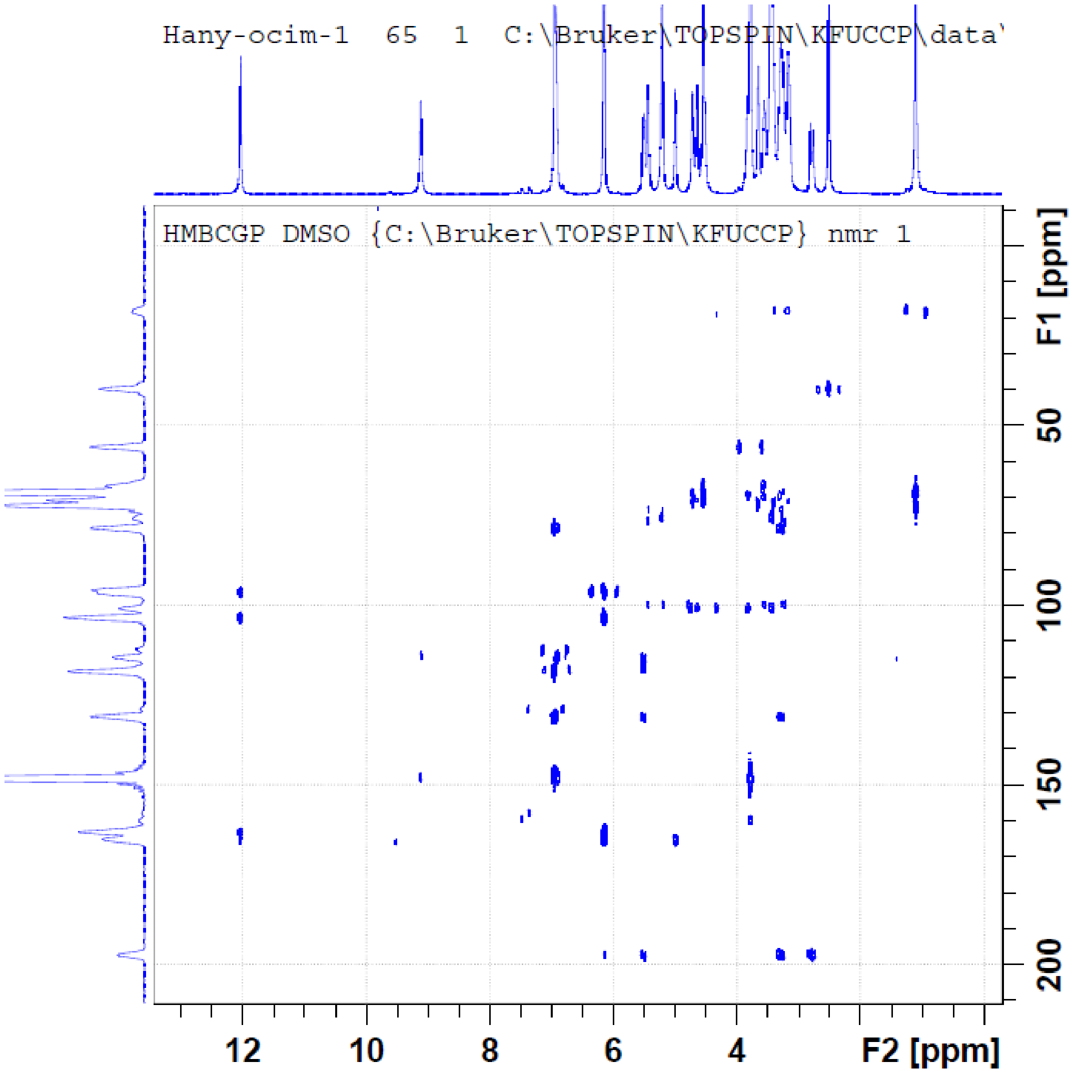

<u>**5- HMBC spectrum of Hesperidin**</u>
