# Supplementary material for: Ameliorative Effect of Ocimum forskolei Benth on Diabetic, Apoptotic, and Adipogenic Biomarkers of Diabetic Rats and 3T3-L1 Fibroblasts Assisted by In Silico Approach
Source: Molecules. 2022 Apr 28;27(9):2800. doi: 10.3390/molecules27092800 (PMC9101318; doi:10.3390/molecules27092800)
Supplement: Supplementary file 1 [file molecules-27-02800-s001.zip › Supplementary‎-S2‎.pdf]

Supplementary-S2

Table 2. Interactions of HSP and amino acid residues of SUR1 receptor.

Binding energy	Ligand efficiency	Intermole energy	Ligand atoms (ring)	Docked amino acid residue (bond length)
-6.75	-0.16	-8.07	- Conventional hydrogen bonds:	
			'Ring - C' C4-O	Chain A: THR`297'HG1'(1.85 Å)
			C2′′′-H	Chain B: ALA`172'O'(2.95 Å)
			C3′′′-H	Chain C: MET`169'O'(2.44 Å)
			C4′′′-H	Chain C: MET`169'O'(2.62 Å)
			C1′′′-O	Chain B: GLY`295'HN'(2.62 Å)
			'Ring - B'C3′-H	Chain B: GLU`288'OE1'(2.83 Å)
			- Carbon hydrogen bonds:	
			'Ring - B'C′-H	Chain B: SER`212'O' (3.71 Å)
			'Ring - B'C′-H	Chain B: GLU`288'O' (3.47 Å)
			C1′′-H	Chain A: GLY`295'O' (3.19 Å)
			- Alkyl hydrophobic interaction:	Chain C: ILE`296' (4.83 Å)
			C6′′′	Chain A: VAL`290' (5.22 Å)
			- Pi-alkyl hydrophobic interaction: O	
			- Miscellaneous sulfur interaction:	Chain C: MET`169''SD' (3.24 Å)
			C4′′′-O	

ALA; Alanine, GLU; Glutamic acid, GLY; Glycine, ILE; Isoleucine, MET; Methionine, SER; Serine, THR; Threonine, VAL; Valine..

Table 3. Interactions of GB and amino acid residues of SUR1 receptor.

Binding energy	Ligand efficiency	Intermole energy	Ligand atoms (ring)	Docked amino acid residue (bond length)
-6.83	-0.21	-9.04	- Conventional hydrogen bonds:	
			C7-O	Chain B: GLY`295 'HN'(1.91 Å)
			C7′-O	Chain D: GLY`295 'HN'(2.43 Å)
			- Carbon hydrogen bonds:	
			C1′-SO	Chain D: THR`294'CB'(2.76 Å)
			C2-OC	Chain C: GLN`173'OE1'(3.20 Å)
			- Alkyl hydrophobic interaction:	
			O	
			OC	Chain A: ALA`172' (3.65 Å)
			OC	Chain C: MET`169' (5.02 Å)
			- Pi-alkyl hydrophobic interaction:	Chain C: ALA`172' (3.50 Å)
			O,	
			O	Chain B: ALA`172' (5.23 Å)
			- Pi-alkyl sigma interaction: O	Chain C: MET`169' (4.98 Å)
				Chain D: ILE`296' CG2' (3.92 Å)

ALA; Alanine, GLN; Glutamine, GLY; Glycine, ILE; Isoleucine, MET; Methionine, THR; Threonine.

Table 4. Interactions of HSP and amino acid residues of NF-κB.

Binding energy	Ligand efficiency	Intermole energy	Ligand atoms (ring)	Docked amino acid residue (bond length)
-4.92	-0.11	-5.61	- Conventional hydrogen bonds:	
			C2′′′-OH	Chain A: ARG`187′O′(2.16 Å)
			C4′′-O	Chain A: LYS`218′HZ1′(1.85 Å)
			C4′′-OH	Chain A: GLN`247′OE1′(1.86 Å)
			C3′-O(B)	Chain B: LYS`572′HZ2′(1.77 Å)
			C3′-OH(B)	Chain B: GLN`606′OE1′(2.27 Å
			- Carbon hydrogen bonds:	
			C1′′′-O	Chain A: LYS`218′CE′(2.80 Å)
			C3′′′-O	Chain A: ARG`187′CD′(3.49 Å)
			- Alkyl hydrophobic interaction: C6′′′	Chain A: LYS`218′(4.16 Å)

ARG; Arginine, GLN; Glutamine, LYS; Lysine.

Table 5. Interactions of GB and amino acid residues of NF-κB.

Binding energy	Ligand efficiency	Intermole energy	Ligand atoms (ring)	Docked amino acid residue (bond length)
-4.70	-0.14	-6.80	- Conventional hydrogen bonds:	
			C2-O	Chain B: LYS`572′HZ3′(2.07 Å)
			C1′-SO	Chain B: GLN`606′HE21′(2.64 Å)
			-Carbon hydrogen bonds : C	Chain B: LYS`541′O′(2.82 Å)
			-Pi-alkyl hydrophobic interaction O :	Chain B: LYS`572′(4.45 Å)
			-Pi-Pi T-shaped hydrophobic interaction:O	Chain B: PHE`607′(4.73 Å)
			-Pi-sulfur interaction: C1′-S	Chain B: Chain B: PHE`607′(4.73 Å)

GLN; Glutamine, LYS; Lysine, PHE; Phenylalanine.

Table 6. Interactions of HSP and amino acid residues of CASPASE-3.

Binding energy	Ligand efficiency	Intermole energy	Ligand atoms (ring)	Docked amino acid residue (bond length)

-8.44	-0.20	-9.34	- Conventional hydrogen bonds:	
			C4′′′-OH	Chain C: THR`255′OG1′(2.30 Å)
			C4′′′-O	Chain A: THR`62′HG1′(2.77 Å)
			C3′′′-OH	Chain A: THR`62′OG1′(2.12 Å)
			C2′′-O	Chain C: LEU`168′HN′(2.26 Å)
			C2′′-OH	Chain C: THR`166′O′(2.34 Å)
			C3′-O (B)	Chain A: LEU`168′HN′(2.69 Å)
			C3′-OH (B)	Chain A: GLU`167′OE1′(1.75 Å)
			- Carbon hydrogen bonds:	
			C2-O (C)	Chain A: THR`166′CB1′(3.61 Å)
			- Pi-alkyl hydrophobic interaction:	
			C6′′′	Chain A: HIS`121′(4.09 Å

GLU; Glutamic acid, HIS; Histidine, LEU; Leucine, THR; Threonine.

Table 7. Interactions of GB and amino acid residues of CASPASE-3.

Binding energy	Ligand efficiency	Intermole energy	Ligand atoms (ring)	Docked amino acid residue (bond length)
-6.35	-0.19	-8.31	- Conventional hydrogen interaction:	
			C1′-SO	Chain A: LEU`168′HN′(2.56 Å)
			C7′-NH	Chain A: THR`166′OG1′(2.16 Å)
			C1′′-NH	Chain A: THR`166′OG1′(1.97 Å)
			- Carbon hydrogen interaction:	
			C1′-SO	Chain A: GLU`167′CA′(3.25 Å)
			C7-O	Chain A: THR`166′CA′(2.81 Å)

GLU; Glutamic acid, LEU; Leucine, THR; Threonine.

Table 8. Interactions of HSP and amino acid residues of BCL2.

Binding energy	Ligand efficiency	Intermole energy	Ligand atoms (ring)	Docked amino acid residue (bond length)
-6.07	-0.14	-6.31	- Conventional hydrogen bonds:	
			C5-OH(A)	Chain A: PHE`144′O′(2.18 Å)
			C3′(B)	Chain A: ASP`95′OD1′(1.93 Å)
			- Pi-anion interaction: O (B)	Chain A: GLU`98′OE1′(4.19 Å)
			- Pi-sulfur interaction: O(A)	Chain A: MET`170′(5.70 Å)

ASP; Aspartic acid, GLU; Glutamic acid, MET; Methionine, PHE; Phenylalanine.

Table 9. Interactions of GB and amino acid residues of BCL2.

Binding energy	Ligand efficiency	Intermole energy	Ligand atoms (ring)	Docked amino acid residue (bond length)

-6.67	-0.20	-8.56	- Conventional hydrogen bonds:	
			C4-NH	Chain A: PHE`144′O′(3.02 Å)
			C1′-SO	Chain A: ARG`91′HE′(2.23 Å)
			C7′-NH	Chain A: LYS`87′O′(3.05 Å)
			- Carbon hydrogen bonds: C2-OH	Chain A: SER`145′OG′(3.47 Å)
			- Alkyl hydrophobic interaction:	
			C4``	Chain A: VAL`86′(5.24 Å)
			C3``	Chain A: TRP`188′(5.08 Å)
			C3`	Chain A: LEU`90′(5.31 Å)
			- Pi-anion interaction: O	Chain A: GLU`98′OE2′(4.35 Å)

ARG; Arginine, GLU; Glutamic Acid, LEU; Leucine LYS; Lysine, PHE; Phenylalanine, SER; Serine, TRP; Tryptophan, VAL; Valine.

Table 10. Interactions of HSP and amino acid residues of DGAT1.

Binding energy	Ligand efficiency	Intermole energy	Ligand atoms (ring)	Docked amino acid residue (bond length)
-7.07	-0.16	-8.46	- Conventional hydrogen bonds:	
			C3′′′-OH	Chain B: LEU`355′O′(2.17 Å)
			C2′′′-OH	Chain B: LEU`355′O′(2.37 Å)
			C2-O (C)	Chain A: CYS`92′SG′(3.01 Å)
			- Pi-donor hydrogen interaction: O	Chain A: CYS`92′SG′(3.66 Å)
			- Alkyl hydrophobic interaction: C4′ (B)	Chain A: LEU`89′(4.09 Å)
				Chain A: TYR`339′(4.11 Å)
			- Pi-sigma hydrophobic interaction:	
			C6′′′	Chain A: TYR`339′(3.48 Å)
			C	Chain B: TRP`91′CZ3′(3.48 Å)
			- Miscellaneous pi-sulfur interaction O :	Chain A: CYS`92′SG′(5.12 Å)

CYS; Cysteine, LEU; Leucine, TRP; Tryptophan, TYR; Tyrosine.

Table 11. Interactions of HSP and amino acid residues of CEBP/α.

Binding energy	Ligand efficiency	Intermole energy	Ligand atoms (ring)	Docked amino acid residue (bond length)
-6.64	-0.15	-7.54	- Conventional hydrogen bonds:	
			C4′′-O	Chain C: ARG`306′HH22′(3.56 Å)
			C1′′-O	Chain C: ASN`307′HD22′(1.94 Å)
			C3′-OH(B)	Chain A: GLN`312′OE1′(2.10 Å)
			- Alkyl hydrophopic interaction: C4′(B)	Chain A: LEU`315′(4.19 Å)
			- Pi-alkyl hydrophopic interaction:	
			O(B)	Chain A: VAL`308′(5.47 Å)
			O(A)	Chain A: VAL`308′(5.15 Å)

ARG; Arginine, ASN; Asparagine, GLN; Glutamine, LEU; Leucine, VAL; Valine.

Table 12. Interactions of HSP and amino acid residues of PPARγ.

Binding energy	Ligand efficiency	Intermole energy	Ligand atoms (ring)	Docked amino acid residue (bond length)
-4.61	-0.11	-8.83	- Conventional hydrogen bonds:	
			Ring - B′C3´-OH	Chain B: GLU`324′OE2′(1.85 Å)
			Ring - B′C3´-O	Chain B: ARG`397′HH22′(2.12 Å)
			C2-O	Chain A: GLN`444′HE21′(2.564 Å)
			C6´´-O	Chain A: GLN`444′HE21′(2.75 Å)
			C2´´´-OH	Chain B: ASP`396′OD1′(1.97 Å)
			C2´´´-OH	Chain B: ASP`396′O′(1.99 Å)
			C3´´-OH	Chain A: PRO`366′O′(2.02 Å)
			- Carbon hydrogen bond: C6´´-H	Chain A: ASP`441′OD1′(3.21 Å)
			- Alkyl hydrophobic interaction: C4´	Chain B: VAL`446′(5.19 Å)
			- Pi-alkyl hydrophobic interaction: C4´	Chain B: TYR`320′ (5.18 Å)
			- Pi-Pi T-shaped interaction: Ring - B ‘O′	Chain B: TYR`320′ (4.67 Å)

ARG; Arginine, ASP; Aspartic acid, GLN; Glutamine, GLU; Glutamic acid, PRO; Proline, TYR; Tyrosine, VAL; Valine.
